# Social and behavioral factors related to blood pressure measurement: A cross-sectional study in Bhutan

**DOI:** 10.1371/journal.pone.0271914

**Published:** 2022-08-17

**Authors:** Hiromi Kohori Segawa, Hironori Uematsu, Nidup Dorji, Ugyen Wangdi, Chencho Dorjee, Pemba Yangchen, Susumu Kunisawa, Ryota Sakamoto, Yuichi Imanaka

**Affiliations:** 1 Department of Healthcare Economics and Quality Management, Graduate School of Medicine, Kyoto University, Kyoto City, Kyoto, Japan; 2 Kokoro Research Center, Kyoto University, Kyoto City, Kyoto, Japan; 3 Faculty of Nursing and Public Health, Khesar Gyalpo University of Medical Sciences of Bhutan, Thimphu, Kingdom of Bhutan; 4 Non-Communicable Diseases Division, Ministry of Health in Bhutan, Thimphu, Kingdom of Bhutan; 5 Centre for Southeast Asian Studies, Kyoto University, Kyoto City, Kyoto, Japan; Faculdade de Medicina de São José do Rio Preto, BRAZIL

## Abstract

Cardiovascular disease is a leading cause of death in the Kingdom of Bhutan, and early detection of hypertension is critical for preventing cardiovascular disease. However, health-seeking behavior, including blood pressure measurement, is infrequently investigated in Bhutan. Therefore, this study investigated factors related to blood pressure measurement in Bhutan. We performed a secondary data analysis of a target population of 1,962 individuals using data from the “2014 Bhutan STEPS survey data”as a cross-sectional study. Approximately 26% of those with hypertension who were detected during the STEPS survey had never had their blood pressure measured. Previous blood pressure measurement was significantly associated with age and working status in men (self-employed [odds ratio (OR): 0.219, 95% CI: 0.133–0.361], non-working [OR: 0.114, 95% CI: 0.050–0.263], employee [OR: 1.000]). Previous blood pressure measurement was significantly associated with higher income in women (Quartile-2 [OR: 1.984, 95% CI: 1.209–3.255], Quartile-1 [OR: 2.161, 95% CI: 1.415–3.299], Quartile-4 [OR: 1.000]). A family history of hypertension (OR: 2.019, 95% CI: 1.549–2.243) increased the likelihood of having experienced a blood pressure measurement in both men and women. Multivariate logistic regression showed that people with unhealthy lifestyles (high salt intake [adjusted odds ratio (AOR): 0.247, 95% confidence interval (CI): 0.068–0.893], tobacco use [AOR: 0.538, 95% CI: 0.380–0.761]) had a decreased likelihood of previous blood pressure measurement. To promote the early detection of hypertension in Bhutan, we suggest that more attention be paid to low-income women, non-working, self-employed, and low-income men, and a reduction of barriers to blood pressure measurement. Before the STEPS survey, a substantial number of hypertensive people had never had their blood pressure measured or were unconcerned about their health. As a result, we propose that early blood pressure monitoring and treatment for people with hypertension or at higher risk of hypertension be given increased emphasis.

## Introduction

According to the World Health Organization (WHO), 38 million people die from non-communicable diseases (NCDs) annually. Approximately three-quarters of these deaths (28 million) occur in low- and middle-income countries [1]. This increase in NCDs, such as cardiovascular disease, has led to an economic burden on individuals, families, and society [2].

Hypertension is one of the strongest risk factors for almost all cardiovascular diseases. The course of cardiovascular disease is indicated by asymptomatic alterations in numerous organs linked with hypertension [3]. Secondary prevention strategies such as screening, early detection, counseling, and continued follow-up of people with high blood pressure are essential to prevent cardiovascular-related diseases. There is, however, a distinction between the true prevalence of hypertension and the rates of diagnosis and treatment [4].

Similarly, in the Kingdom of Bhutan, mortality due to NCDs has increased from 53% (2008) to 69% (2016) [5, 6] and is the leading cause of mortality [6]. The government of Bhutan provides free access to basic public health services for all its citizens, and the Ministry of Health has initiated primary prevention programs such as maintaining a healthy diet, avoiding alcohol and tobacco, and exercising, all of which are essential in preventing NCDs [7–9].

However, a previous qualitative study indicated that most Bhutanese have inadequate knowledge about how to protect themselves from hypertension through daily practices, although they wish to be healthy because health is an important factor for happiness [10]. The participants were diagnosed with hypertension during previous fieldwork conducted in 2017, and some had never had their blood pressure measurement before this fieldwork and were unaware of the importance of regular blood pressure measurement [10]. Furthermore, according to the STEPS survey report, “Around 31.3% of the study population had never had their blood pressure checkup. The prevalence of raised blood pressure or hypertension (SBP≥140 and/or DBP≥90) was 35.7% (men 35.5%, women 35.9%) when those currently using medication were included.” [7].

Blood pressure measurement is vital as a first step in subsequent hypertension prevention. Therefore, the STEPS survey data can be used to determine which social backgrounds are associated with lower blood pressure measurements in Bhutan. This can also reveal which high-risk behaviors for hypertension are associated with blood pressure measurement. However, in Bhutan, these topics have received less attention, especially in terms of quantitative and representative research.

In this study, we looked into the social backgrounds of those who had less access to blood pressure measurement, as well as behavioral factors associated with less blood pressure measurement experience, in order to contribute to more hypertension prevention efforts in Bhutan.

We need to consider both aspects of " access and behaviors" and "vulnerability" when examining the prevention of severe hypertension and early detection of hypertension. We have a related manuscript, the aspect from "vulnerability". https://doi.org/10.1371/journal.pone.0256811.

### Objectives

To use the STEPS survey in Bhutan to investigate

1. The social factors that are associated with less blood pressure measurement experience.
2. Whether high-risk behavioral factors for hypertension are associated with less blood pressure measurement experience.

## Methods

### Study design

This cross-sectional study was conducted using data from the “National Survey for Noncommunicable Disease Risk Factors and Mental Health using WHO STEPS Approach in Bhutan– 2014” [7].

### Study setting

Bhutan is located in the eastern Himalayas between India in the east, west, and south and China in the north. This region has an area of 38,394 km^2^. As of 2020, the Bhutanese population was estimated to be 748,931 [11]. The gross domestic product (GDP) per capita was estimated to be USD 3,411.94 in 2019 [11]. Bhutan’s national development is based on the philosophy of “gross national happiness (GNH)”, which aspires to sustainable development and happiness for all its citizens [12, 13].

### Data sources

Data were derived from the “National Survey for Noncommunicable Disease Risk Factors and Mental Health using WHO STEPS Approach in Bhutan– 2014”, which was conducted by WHO and the government of Bhutan from March to June 2014 [7]. The sample size was 2,912, which was considered sufficient to represent the target population (adults aged 18–69 years) in Bhutan (S1 File). To ensure the representativeness of the sample, the study applied multistage cluster sampling combined with probability measures proportionate to the size and systematic random sampling. The strata level considered was urbanicity (rural: urban = 7:3); an area block was designated as the cluster level (n = 182) and was selected from *gewogs* (group of villages). The Kish method was applied to select participants from each household using age and gender as variables, and the sampling framework from the “Population and Housing Census of Bhutan 2005” was employed [14]. According to the sampling plan, trained health staff recruited participants in each sampling field where the participants were living. Trained health staff collected data through face-to-face interviews. The number of valid respondents was 2,822 with a response rate of 97%. A previous report by WHO and the Ministry of Health in Bhutan provides the details of the survey procedure [7].

### Definitions of variables

#### Objective variables

Previous blood pressure measurement was defined as 1 for "Yes" and 0 for "No" for the questionnaire item in the survey “Have you ever had your blood pressure measured by a doctor or other health worker?” (This question regarded the period before the survey, excluding blood pressure measurement that occurred during the survey).

#### Explanatory variables

Explanatory variables were selected and categorized mainly by following the WHO guidelines [15] and previous literature [16–30].

Gender (men, women), marital status (married or cohabiting, never married, separated or divorced or widowed), age, level of education (non-formal education, elementary education [1–10 years], high school education [11–12 years], and tertiary education [above 12 years]; a promotion test is given in the 10th and 12th years in Bhutan), working status (i.e., employed, self-employed, and non-working), residential area (urbanicity), income (Quartile-1 (Q1) (Nu. 60,001+: USD 805.8+), Quartile-2 (Q2) (Nu. 30,001–60,000: USD 403.0–805.7), Quartile-3 (Q3) (Nu. 9,001–30,000: USD 120.9–402.9), Quartile-4 (Q4) (Nu. 0–9,000: USD 0–120.8)), and survey language (Dzongkha, Tshanglakha, Lhotshamkha, English) were also recorded.

Tobacco use (never, current, or any [not only smoking but also chewing tobacco]) [15], alcohol consumption (none, light or moderate, or heavy) [15, 21], fruit and vegetables consumption (<5, 5, or >5 portions per day) [15, 19], physical activity level (≥150 min of moderate activity per week or <150 min of moderate activity per week) [15, 20], and estimated salt intake (<5 g, 5 g, or >5 g per day with a cutoff point determined according to the WHO guidelines and the Tanaka formula used for estimation: Salt intake per day(g) = (21.98 × ((spot urinary sodium/ (spot urinary creatinine × 10/0.0884)) × (((14.89 × weight [Kg]) +(16.14 × height [m]) × (2.04 × age [years])) - 2244.45)) × 0.392)/17.1) were investigated [15, 22–26]. All variables were measured using the WHO guidelines.

Hypertension was defined by the following criteria: (1) systolic blood pressure (SBP) ≥140 mmHg or diastolic blood pressure (DBP) ≥90 mmHg measured as the mean of three measurements taken by health staff in the STEPS survey; (2) a previous diagnosis of hypertension by healthcare workers; (3) current treatment with medication for hypertension [15].

### Statistical methods

Descriptive statistics were used to describe the characteristics of the study population. Univariable logistic regression was performed to evaluate the associations between objective variables and each explanatory variable. All available and selected data from the STEPS survey were considered to be related to the objective variables from previous literature. Among those variables that may be related to blood pressure measurement, we selected explanatory variables to be fed into the model using a directed acyclic graph (S2 File).

Health behavior such as blood pressure measurement is affected by various factors. In particular, we considered both individual and social environmental factors as targets for health promotion interventions. “Interpersonal” processes are known to shape human behavior and lifestyles [25, 26].

Health policy is common for all Bhutanese across the country. Some but not all of the primary prevention programs for NCDs, such as displays of posters in health facilities, had already started by 2014 at the time of the survey. The number of primary health centers (PHCs) was also increasing in rural areas due to the expansion of primary health care. We predict that the situations of diagnosis and risk perception were also diverse. Furthermore, risk perception (threat) is also known to affect human behavior [3]. We selected individual and social environmental factors that influence interpersonal decisions or close networks. Gender, marital status, age, level of education, working status, income, and survey language were among the selected characteristics. Tobacco use, alcohol consumption, fruit and vegetable consumption, physical activity level, estimated salt intake, history of hypertension, and family history of hypertension were all chosen as lifestyle-related and biological risk perception factors.

We evaluated the association between previous blood pressure measurement and the individual and social environmental factors affecting interpersonal decisions or close networks through descriptive and univariable logistic regression, the association between previous blood pressure measurement and the high-risk behavioral factors of hypertension through descriptive and multivariable logistic regression (S2 File: Directed acyclic graph for multivariable logistic regression). Through the directed acyclic graph, we defined the minimal sufficient adjustment sets for estimating the total effect of high-risk behavioral factors of hypertension on the experience of having a blood pressure measurement.

All data were analyzed using IBM Statistical Package for Social Sciences version 23 with the module for Complex Sample Analysis (IBM Corp., Armonk, NY, USA), excluding the non-weighted calculation for Table 1 (mean and standard deviation). All estimates are presented with 95% confidence intervals (CIs) after adjusting for weight following the WHO guidelines to ensure they were representative of the national population [7] and adjusted based on the adjusted F-value, which is a variant of the second-order Rao–Scott adjusted chi-square statistic given the multistage random sampling [31, 32]. Variables with a *p*-value <0.05 were considered significant. In the study, participants aged 18–69 years were included. Those with missing data were excluded, and pregnant women were excluded because of health behavioral and biological differences.

**Table 1:** Distribution of socio-demographic characteristics and life behaviors.

		n	Mean^1)^	Standard deviation^1)^	Weight adjusted^2)^	BP measured^3)^	Estimated^4)^	95% CI				
Total		1962			100%	1402	67.1%	(	62.9%	–	71.2%	)	
Gender	Men	772			57.6%	486	60.8%	(	54.5%	–	66.8%	)	
	Women	1190			42.4%	916	75.8%	(	72.4%	–	78.9%	)	
Marital status	Married or cohabiting	1588			83.1%	1158	69.6%	(	64.9%	–	73.8%	)	
	Never married	155			10.4%	86	46.7%	(	37.2%	–	56.4%	)	
	Separated, divorced, or widowed	219			6.5%	158	69.1%	(	59.9%	–	77.0%	)	
Age			40.25	12.36									
	18–29 years	443			28.5%	297	61.4%	(	54.7%	–	67.7%	)	
	30–39 years	571			36.6%	427	67.7%	(	61.3%	–	73.6%	)	
	40–49 years	475			17.1%	349	73.3%	(	67.9%	–	78.2%	)	
	50–59 years	306			11.4%	213	68.6%	(	61.2%	–	75.2%	)	
	60–69 years	167			6.3%	116	70.0%	(	60.3%	–	78.2%	)	
Education			4.90	12.05									
	No formal education	1203			54.1%	828	63.6%	(	58.4%	–	68.5%	)	
	1–10 years	600			35.9%	453	71.2%	(	65.8%	–	76.0%	)	
	11–12 years	99			6.2%	70	63.0%	(	49.0%	–	75.1%	)	
	Above 12 years	60			3.7%	51	86.5%	(	72.6%	–	94.0%	)	
Working status	Employee	342			23.7%	278	82.7%	(	76.5%	–	87.5%	)	
	Self-employed	1081			53.3%	746	62.6%	(	58.6%	–	66.4%	)	
	Non-working	539			23.0%	378	61.7%	(	50.7%	–	71.6%	)	
Income			924.61	3164.90									
	USD 0–120.8	502			25.2%	325	58.5%	(	51.3%	–	65.3%	)	
	USD 120.9–402.9	546			29.3%	360	58.2%	(	51.6%	–	64.5%	)	
	USD 120.9–402.9	412			22.7%	319	78.2%	(	71.7%	–	83.5%	)	
	USD 403.0–805.7	502			22.8%	398	77.2%	(	71.5%	–	82.0%	)	
Survey language	Dzongkha	701			33.8%	514	71.8%	(	66.6%	–	76.6%	)	
	Tshanglakha	680			30.8%	494	70.7%	(	63.3%	–	77.2%	)	
	Lhotshamkha	547			33.2%	368	58.6%	(	50.8%	–	66.1%	)	
	English	34			2.2%	26	73.4%	(	51.0%	–	87.9%	)	
Family history of hypertension	Negative	1311			64.9%	882	62.0%	(	57.0%	–	66.7%	)	
	Positive	651			35.1%	520	76.7%	(	71.9%	–	80.9%	)	
Tobacco use	Never use	1556			74.3%	1151	71.0%	(	66.6%	–	75.0%	)	
	Currently use	406			25.7%	251	56.1%	(	48.6%	–	63.4%	)	
Alcohol consumption	Never drink	990			47.7%	721	67.8%	(	62.6%	–	72.6%	)	
	Light or moderate drinker	548			30.3%	379	66.7%	(	59.1%	–	73.6%	)	
	Heavy drinker	424			21.9%	302	66.3%	(	59.2%	–	72.8%	)	
Fruit and vegetables consumption			4.52	3.48									
	Above 5 portions per day	656			33.9%	485	69.3%	(	62.0%	–	75.8%	)	
	Below or 5 portions per day	1306			66.1%	917	66.0%	(	61.3%	–	70.5%	)	
Physical activity			475.97	682.04									
	Above or 150 mins per week	1811			93.3%	1295	67.0%	(	62.5%	–	71.2%	)	
	Below 150 mins per week	151			6.7%	107	69.2%	(	58.9%	–	77.8%	)	
Salt intake			14.42	12.81									
	Below 5 g per day	22			0.8%	18	86.1%	(	65.2%	–	95.3%	)	
	Above or 5 g per day	1940			99.2%	1384	67.0%	(	62.7%	–	71.0%	)	
Blood pressure^5)^	Normal	1068			58.0%	705	62.4%	(	57.0%	–	67.5%	)	
	High	894			42.0%	697	73.7%	(	68.6%	–	78.3%	)	

^1)^ Non-weighted calculation

^2)^ Complex sampling weight-adjusted %

^3)^ Participants who answered "Yes" to the questionnaire item “Have you ever had your blood pressure measured by a doctor or other health worker?”

^4)^ Estimated % adjusted with complex sampling: Participants with a previous blood pressure measurement/total target population

^5)^ "High" blood pressure was defined by the following criteria: (1) systolic blood pressure (SBP) ≥140 mmHg or diastolic blood pressure (DBP) ≥90 mmHg, measured as the mean of three measurements taken by health staff in the STEPS survey; (2) a previous diagnosis of hypertension by healthcare workers; (3) currently taking medication for hypertension.

In addition, given the gender differences and mother-child health programs, we also conducted a subgroup analysis for gender groups.

## Research ethics

The study protocol was approved by the Research Ethics Board of Health, Ministry of Health of the Royal Government of Bhutan (No. REBH/Approval/2018/089) and the Ethics Committee of the Kyoto University Hospital and Graduate School of Medicine (No. R1796). The above ethics committees waived the requirement for individual informed consent because this secondary analysis used only de-identified data. We have submitted a written pledge of confidentiality to the Ministry of Health of the Royal Government of Bhutan.

## Results

**Fig 1:**
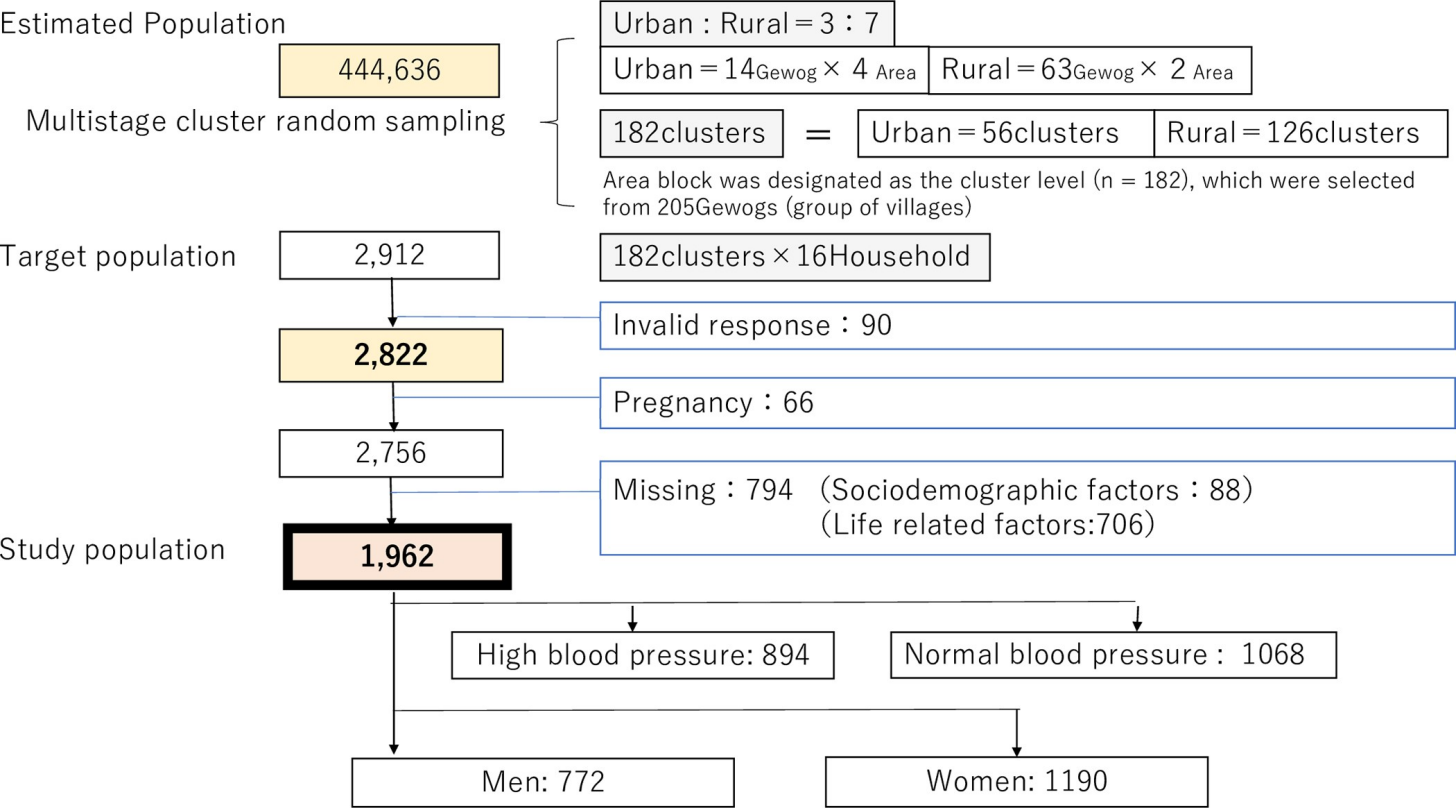
Target population.

Our target participants included 1,962 individuals (Fig 1).

Table 1 shows the distribution of sociodemographic characteristics of all participants in this study. The target participants included 772 men (57.6%, standard error (SE): 1.9%, 95% CI: 53.7–61.4%) and 1,190 women (42.4%, SE: 1.9%, 95% CI: 38.6–46.3%). Most participants were married or cohabiting (83.1%, SE: 1.2%, 95% CI: 80.5–85.4%). The mean participant age was 40.25 years (non-weighted), 37.5 years (weighted) (SD: 12.4, SE: 0.407, 95% CI: 36.7–38.3). Over half of participants had no education (54.1%, SE: 2.1%, 95% CI: 50.0–58.2%) and were self-employed (53.3%, SE: 3.3%, 95% CI: 46.9–59.7%). Forty-two percent (SE: 1.7%, 95% CI: 38.7–45.4%) of all participants had hypertension, and 67.1% (SE: 2.1%, 95% CI: 62.9–71.2%) of participants had never had a blood pressure measurement. Among those with hypertension, 26.3% (crude n = 197, SE: 2.5%, 95% CI: 21.7–31.4%) had never had a blood pressure measurement before.

Table 2 shows the descriptive differences between those who had hypertension or not. In total, those who had hypertension had a higher coverage of blood pressure measurement. However, except for the category of salt intake (<5 g per day), no category had 100% coverage of blood pressure measurement before the NCD STEPS survey.

**Table 2:** Differences between participants with hypertension or not and the distribution of socio-demographic characteristics and life behaviors for blood pressure measurement.

		Hypertension^1)^								Normal blood pressure							
		n	BP measured^2)^	Estimated^3)^	95% CI					n	BP measured^2)^	Estimated^3)^	95% CI				
Gender	Men	348	246	67.6%	(	59.9%	–	74.5%	)	424	240	56.1%	(	48.3%	–	63.6%	)
	Women	548	452	81.4%	(	77.0%	–	85.1%	)	642	464	71.4%	(	66.3%	–	76.0%	)
Marital Status	Married or cohabiting	735	576	74.5%	(	32.9%	–	74.5%	)	115	62	43.8%	(	32.8%	–	55.5%	)
	Never married	40	24	54.5%	(	69.1%	–	79.2%	)	853	582	65.8%	(	59.6%	–	71.5%	)
	Separated, divorced, or widowed	121	98	81.5%	(	70.5%	–	89.0%	)	98	60	55.7%	(	42.0%	–	68.7%	)
Age	18–29 years	98	79	75.2%	(	62.2%	–	84.9%	)	345	218	57.5%	(	50.4%	–	64.3%	)
	30–39 years	238	187	68.4%	(	59.0%	–	76.5%	)	333	240	67.3%	(	59.3%	–	74.4%	)
	40–49 years	263	207	78.2%	(	71.3%	–	83.7%	)	212	142	67.9%	(	59.9%	–	75.0%	)
	50–59 years	186	141	75.7%	(	65.4%	–	83.8%	)	120	72	56.7%	(	46.5%	–	66.3%	)
	60–69 years	111	84	77.7%	(	66.7%	–	85.8%	)	56	32	53.1%	(	37.4%	–	68.3%	)
Education	No formal education	611	470	72.6%	(	65.9%	–	78.5%	)	592	358	55.5%	(	48.5%	–	62.2%	)
	1–10 years	243	194	73.9%	(	65.1%	–	81.1%	)	357	259	69.5%	(	62.7%	–	75.6%	)
	11–12 years	18	14	75.6%	(	43.3%	–	92.6%	)	81	56	60.0%	(	44.2%	–	74.0%	)
	Above 12 years	24	20	90.2%	(	73.5%	–	96.8%	)	36	31	84.2%	(	63.2%	–	94.3%	)
Working status	Employee	142	125	88.3%	(	81.0%	–	93.0%	)	200	153	79.1%	(	70.5%	–	85.7%	)
	Self-employed	524	392	68.4%	(	62.1%	–	74.0%	)	557	354	57.9%	(	52.4%	–	63.2%	)
	Non-working	230	181	73.1%	(	60.7%	–	82.7%	)	309	197	54.4%	(	43.4%	–	64.9%	)
Income	USD 0–120.8	235	167	64.2%	(	55.5%	–	72.1%	)	267	158	53.6%	(	45.0%	–	62.1%	)
	USD 120.9–402.9	262	200	68.6%	(	58.6%	–	77.2%	)	284	160	50.5%	(	42.8%	–	58.2%	)
	USD 120.9–402.9	177	150	88.9%	(	81.5%	–	93.5%	)	235	169	72.2%	(	62.4%	–	80.3%	)
	USD 403.0–805.7	222	181	78.5%	(	70.5%	–	84.9%	)	280	217	76.1%	(	68.6%	–	82.3%	)
Survey language	Dzongkha	279	224	78.0%	(	69.7%	–	84.6%	)	422	290	67.5%	(	59.9%	–	74.4%	)
	Tshanglakha	369	281	73.8%	(	65.0%	–	81.0%	)	311	213	67.9%	(	58.1%	–	76.3%	)
	Lhotshamkha	243	189	68.8%	(	58.7%	–	77.4%	)	304	179	52.0%	(	43.7%	–	60.1%	)
	English	5	4	87.5%	(	39.0%	–	98.7%	)	29	22	71.3%	(	46.6%	–	87.6%	)
Family history of hypertension	Negative	580	427	68.5%	(	61.5%	–	74.7%	)	731	455	57.4%	(	51.5%	–	63.0%	)
	Positive	316	271	83.1%	(	76.9%	–	87.9%	)	335	249	71.9%	(	64.7%	–	78.0%	)
Tobacco use	Never use	733	579	75.9%	(	70.6%	–	80.5%	)	823	572	67.2%	(	61.7%	–	72.3%	)
	Currently use	163	119	66.6%	(	55.8%	–	75.9%	)	243	132	49.7%	(	40.5%	–	59.0%	)
Alcohol consumption	Never drink	403	327	75.2%	(	68.8%	–	80.7%	)	587	394	63.2%	(	56.7%	–	69.2%	)
	Light or moderate drinker	258	190	69.9%	(	60.3%	–	78.0%	)	290	189	64.3%	(	54.5%	–	73.1%	)
	Heavy drinker	235	181	76.0%	(	68.0%	–	82.4%	)	189	121	57.3%	(	46.8%	–	67.3%	)
Fruit and vegetables consumption	Above 5 portions per day	319	245	71.2%	(	61.9%	–	79.0%	)	337	240	67.8%	(	58.2%	–	76.1%	)
	Below or 5 portions per day	577	453	75.1%	(	69.5%	–	80.0%	)	729	464	59.7%	(	53.9%	–	65.2%	)
Physical activity	Above or 150 mins per week	832	649	73.8%	(	68.3%	–	78.7%	)	979	646	62.0%	(	56.5%	–	67.3%	)
	Below 150 mins per week	64	49	72.5%	(	57.4%	–	83.7%	)	87	58	67.0%	(	53.3%	–	78.3%	)
Salt intake	Below 5 g per day	10	10	100.0%	(	100.0%	–	100.0%	)	12	8	76.5%	(	46.1%	–	92.5%	)
	Above or 5 g per day	886	688	73.5%	(	68.3%	–	78.1%	)	1054	696	62.2%	(	56.8%	–	67.4%	)

^1)^ Hypertension was defined by the following criteria: (1) systolic blood pressure (SBP) ≥140 mmHg or diastolic blood pressure (DBP) ≥90 mmHg, measured as the mean of three measurements taken by health staff in the STEPS survey; (2) a previous diagnosis of hypertension by healthcare workers; (3) currently taking medication for hypertension.

^2)^ Participants who answered "Yes" to the questionnaire item “Have you ever had your blood pressure measured by a doctor or other health worker?”

^3)^ Estimated % adjusted with complex sampling: Participants with a previous blood pressure measurement/total target population

Among those who had normal blood pressure, current tobacco use, heavy alcohol drinker, less fruit and vegetables consumption, and high salt intake had less coverage of blood pressure measurement. However, a shortage of physical activity had a higher coverage of blood pressure measurement.

Table 3 shows the descriptive differences between men and women.

**Table 3:** Gender differences in the distribution of the socio-demographic characteristics and life behaviors for blood pressure measurement.

		Men								Women							
		n	BP measured^1)^	Estimated^2)^	95% CI					n	BP measured^1)^	Estimated^2)^	95% CI				
Marital status	Married or cohabiting	652	423	63.2%	(	56.5%	–	69.4%	)	936	735	78.6%	(	74.9%	–	81.8%	)
	Never married	80	41	45.0%	(	33.2%	–	57.3%	)	75	45	50.4%	(	35.8%	–	64.8%	)
	Separated, divorced, or widowed	40	22	58.5%	(	38.8%	–	75.8%	)	179	136	73.3%	(	63.8%	–	81.1%	)
Age	18–29 years	142	69	48.9%	(	38.1%	–	59.7%	)	301	228	74.6%	(	68.3%	–	80.0%	)
	30–39 years	223	152	63.1%	(	53.9%	–	71.4%	)	348	275	75.8%	(	70.1%	–	80.7%	)
	40–49 years	192	130	71.0%	(	63.4%	–	77.6%	)	283	219	76.3%	(	68.7%	–	82.4%	)
	50–59 years	137	84	61.3%	(	52.1%	–	69.8%	)	169	129	78.1%	(	68.4%	–	85.5%	)
	60–69 years	78	51	66.0%	(	53.3%	–	76.7%	)	89	65	76.2%	(	62.7%	–	85.9%	)
Education	No formal education	383	214	52.1%	(	43.7%	–	60.3%	)	820	614	74.4%	(	70.3%	–	78.0%	)
	1–10 years	297	205	67.9%	(	60.5%	–	74.6%	)	303	248	77.9%	(	71.1%	–	83.5%	)
	11–12 years	50	31	55.3%	(	38.4%	–	71.1%	)	49	39	80.0%	(	63.4%	–	90.3%	)
	Above 12 years	42	36	87.2%	(	69.6%	–	95.3%	)	1	15	84.0%	(	58.6%	–	95.1%	)
Working status	Employee	246	198	83.2%	(	76.0%	–	88.6%	)	96	80	79.9%	(	66.2%	–	89.0%	)
	Self-employed	424	239	52.1%	(	46.2%	–	57.9%	)	657	507	76.5%	(	72.6%	–	80.0%	)
	Non-working	102	49	36.1%	(	22.5%	–	52.5%	)	437	329	73.7%	(	66.9%	–	79.6%	)
Income	USD 0–120.8	179	94	48.7%	(	38.5%	–	59.0%	)	323	231	70.0%	(	64.1%	–	75.2%	)
	USD 120.9–402.9	219	123	49.2%	(	39.4%	–	59.0%	)	327	237	70.6%	(	64.1%	–	76.4%	)
	USD 120.9–402.9	167	119	75.5%	(	66.6%	–	82.7%	)	245	200	82.2%	(	75.1%	–	87.6%	)
	USD 403.0–805.7	207	150	72.8%	(	63.6%	–	80.3%	)	295	248	83.4%	(	78.2%	–	87.6%	)
Survey language	Dzongkha	259	184	70.2%	(	62.4%	–	76.9%	)	442	330	73.9%	(	68.7%	–	78.6%	)
	Tshanglakha	238	152	65.0%	(	52.9%	–	75.4%	)	442	342	77.2%	(	71.5%	–	82.1%	)
	Lhotshamkha	255	135	48.2%	(	38.8%	–	57.8%	)	292	233	76.1%	(	68.2%	–	82.5%	)
	English	20	15	70.6%	(	42.0%	–	88.8%	)	14	11	83.3%	(	46.5%	–	96.6%	)
Family history of hypertension	Negative	528	307	55.0%	(	47.9%	–	61.9%	)	783	575	71.8%	(	67.2%	–	76.1%	)
	Positive	244	179	72.0%	(	64.0%	–	78.8%	)	407	341	82.7%	(	77.2%	–	87.1%	)
Tobacco use	Never use	525	347	64.4%	(	57.1%	–	71.0%	)	1031	804	77.9%	(	74.2%	–	81.1%	)
	Currently use	247	139	53.8%	(	45.0%	–	62.3%	)	159	112	63.6%	(	53.5%	–	72.6%	)
Alcohol consumption	Never drink	287	183	58.9%	(	50.0%	–	67.2%	)	703	538	75.2%	(	70.8%	–	79.2%	)
	Light or moderate drinker	278	172	63.9%	(	54.0%	–	72.9%	)	270	207	73.2%	(	65.9%	–	79.4%	)
	Heavy drinker	207	131	59.1%	(	50.3%	–	67.3%	)	217	171	81.0%	(	73.1%	–	87.0%	)
Fruit and vegetables consumption	Above 5 portions per day	270	170	63.3%	(	51.8%	–	73.4%	)	386	315	79.1%	(	72.4%	–	84.6%	)
	Below or 5 portions per day	502	316	59.4%	(	52.8%	–	65.6%	)	804	601	74.3%	(	70.1%	–	78.2%	)
Physical activity	Above or 150 mins per week	733	460	60.7%	(	54.1%	–	66.9%	)	1078	835	76.2%	(	72.6%	–	79.4%	)
	Below 150 mins per week	39	26	62.4%	(	43.0%	–	78.4%	)	112	81	72.7%	(	61.6%	–	81.5%	)
Salt intake	Below 5 g per day	10	8	81.3%	(	45.0%	–	95.9%	)	12	10	90.5%	(	65.5%	–	97.9%	)
	Above or 5 g per day	762	478	60.6%	(	54.3%	–	66.6%	)	1178	906	75.6%	(	72.2%	–	78.8%	)
Blood pressure^3)^	Normal	424	240	56.1%	(	48.3%	–	63.6%	)	642	464	71.4%	(	66.3%	–	76.0%	)
	High	348	246	67.6%	(	59.9%	–	74.5%	)	548	452	81.4%	(	77.0%	–	85.1%	)

^1)^ Participants who answered "Yes" to the questionnaire item “Have you ever had your blood pressure measured by a doctor or other health worker?”

^2)^ Estimated % adjusted with complex sampling: Participants with a previous blood pressure measurement/total target population

In total, women had an increased likelihood of previous blood pressure measurements than men.

Table 4 shows the univariable regression for blood pressure measurement for all participants. Women (odds ratio (OR): 2.019, 95% CI: 1.533–2.660) and those who were married or cohabiting (OR: 2.611, 95% CI: 1.731–3.937) or separated, divorced, or widowed (OR: 2.556, 95% CI: 1.439–4.540) had a significantly increased likelihood of previous blood pressure measurement. Those with an education level of 1–10 years (OR: 1.414, 95% CI: 1.104–1.811) also had an increased likelihood of previous blood pressure measurement compared with those with no formal education (OR: 1.000). Regarding working status, self-employed (OR: 0.351, 95% CI: 0.234–0.526) and non-working (OR: 0.338, 95% CI: 0.193–0.593) participants had a decreased likelihood of previous blood pressure measurement than employees (OR: 1.000). In terms of income, all participants in Quartile-4 (Nu. 60,001+: USD 805.8+ (OR: 2.404, 95% CI: 1.575–3.667)) and Quartile-3 (Nu. 30,001–60,000: USD 403.0–805.7 (OR: 2.546, 95% CI: 1.640–3.955)) had an increased likelihood of previous blood pressure measurement compared with those in the lowest income category, Quartile-1 (Nu. 0–9,000: USD 0–120.8 [OR: 1.000]). We found a significant association between Quartile-3, Quartile-4, and previous blood pressure measurement but not between Q2 (Nu. 9,001–30,000: USD 120.9–402.9) and previous blood pressure measurement.

**Table 4:** Univariable logistic regression of previous blood pressure measurement in the target population.

		Total							Men							Women						
		OR	95% CI					*P*-value	OR	95% CI					P-value	OR	95% CI					*P*-value
Gender	Men	Ref	(		–		)	**0.000**	Ref	(	–	–	–	)	–	Ref	(	–	–	–	)	–
	Women	**2.019**	(	1.533	–	2.660	)		–	(	–	–	–	)	–	–	(	–	–	–	)	–
Marital Status	Never married	Ref	(		–		)	**0.000**	Ref	(		–		)	**0.010**	Ref	(		–		)	**0.000**
	Married or cohabiting	**2.611**	(	1.731	–	3.937	)		**2.101**	(	1.253	–	3.524	)		**3.611**	(	1.906	–	6.842	)	
	Separated, divorced, or widowed	**2.556**	(	1.439	–	4.540	)		**1.724**	(	0.669	–	4.441	)		**2.708**	(	1.244	–	5.896	)	
Age	18–29 years	Ref	(		–		)	**0.038**	Ref	(		–		)	**0.004**	Ref	(		–		)	0.968
	30–39 years	1.318	(	0.971	–	1.789	)		**1.790**	(	1.114	–	2.875	)		1.068	(	0.726	–	1.57	)	
	40–49 years	**1.726**	(	1.249	–	2.385	)		**2.559**	(	1.507	–	4.348	)		1.094	(	0.664	–	1.803	)	
	50–59 years	1.371	(	0.909	–	2.069	)		1.658	(	0.958	–	2.871	)		1.216	(	0.683	–	2.168	)	
	60–69 years	1.466	(	0.905	–	2.375	)		**2.032**	(	1.058	–	3.900	)		1.089	(	0.548	–	2.164	)	
Education	No formal education	Ref	(		–		)	**0.008**	Ref	(		–		)	**0.000**	Ref	(		–		)	0.575
	1–10 years	**1.414**	(	1.104	–	1.811	)		**1.951**	(	1.376	–	2.765	)		1.218	(	0.812	–	1.826	)	
	11–12 years	0.975	(	0.534	–	1.778	)		1.140	(	0.539	–	2.410	)		1.382	(	0.577	–	3.314	)	
	Above12 years	**3.677**	(	1.483	–	9.119	)		**6.282**	(	2.029	–	19.452	)		1.805	(	0.494	–	6.603	)	
Working status	Employee	Ref	(		–		)	**0.000**	Ref	(		–		)	**0.000**	Ref	(		–		)	0.587
	Self-employed	**0.351**	(	0.234	–	0.526	)		**0.219**	(	0.133	–	0.361	)		0.820	(	0.406	–	1.656	)	
	Non-working	**0.338**	(	0.193	–	0.593	)		**0.114**	(	0.050	–	0.263	)		0.707	(	0.324	–	1.545	)	
Income	USD 0–120.8	Ref	(		–		)	**0.000**	Ref	(		–		)	**0.000**	Ref	(		–		)	**0.001**
	USD 120.9–402.9	0.989	(	0.714	–	1.372	)		1.021	(	0.606	–	1.718	)		1.033	(	0.710	–	1.502	)	
	USD 120.9–402.9	**2.546**	(	1.640	–	3.955	)		**3.250**	(	1.768	–	5.974	)		**1.984**	(	1.209	–	3.255	)	
	USD 403.0–805.7	**2.404**	(	1.575	–	3.667	)		**2.816**	(	1.524	–	5.203	)		**2.161**	(	1.415	–	3.299	)	
Tobacco use	Never use	Ref	(		–		)	**0.000**	Ref	(		–		)	**0.035**	Ref	(		–		)	**0.003**
	Currently use	**0.523**	(	0.378	–	0.724	)		**0.643**	(	0.427	–	0.969	)		**0.496**	(	0.314	–	0.785	)	
Alcohol consumption	Never drink	Ref	(		–		)	0.925	Ref	(		–		)	0.586	Ref	(		–		)	0.280
	Light or moderate drinker	0.953	(	0.662	–	1.372	)		1.239	(	0.754	–	2.036	)		0.897	(	0.600	–	1.342	)	
	Heavy drinker	0.936	(	0.663	–	1.322	)		1.008	(	0.652	–	1.556	)		1.405	(	0.849	–	2.326	)	
Fruit and vegetables consumption	Above 5 portions per day	Ref	(		–		)	0.408	Ref	(		–		)	0.525	Ref	(		–		)	0.222
	Below or 5 portions per day	0.862	(	0.605	–	1.228	)		0.848	(	0.509	–	1.413	)		0.764	(	0.496	–	1.178	)	
Physical activity	Above or 150 mins per week	Ref	(		–		)	0.681	Ref	(		–		)	0.871	Ref	(		–		)	0.488
	Below 150 mins per week	1.104	(	0.687	–	1.775	)		1.072	(	0.461	–	2.492	)		0.832	(	0.493	–	1.403	)	
Salt intake	Below 5 g per day	Ref	(		–		)	0.066	Ref	(		–		)	0.218	Ref	(		–		)	0.175
	Above or 5 g per day	0.328	(	0.100	–	1.079	)		0.354	(	0.067	–	1.859	)		0.327	(	0.065	–	1.651	)	
Family history of hypertension	Negative	Ref	(		–		)	**0.000**	Ref	(		–		)	**0.000**	Ref	(		–		)	**0.004**
	Positive	**2.019**	(	1.549	–	2.633	)		**2.107**	(	1.427	–	3.112	)		**1.87**	(	1.227	–	2.849	)	
High blood pressure^1)^	Normal	Ref	(		–		)	**0.000**	Ref	(		–		)	**0.011**	Ref	(		–		)	N/A
	High	**1.695**	(	1.280	–	2.243	)		**1.639**	(	1.122	–	2.394	)			(		–		)	

^1)^ "High" blood pressure was defined by the following criteria: (1) systolic blood pressure (SBP) ≥140 mmHg or diastolic blood pressure (DBP) ≥90 mmHg, measured as the mean of three measurements taken by health staff in the STEPS survey; (2) a previous diagnosis of hypertension by healthcare workers; (3) currently taking medication for hypertension.

N/A: Not able to calculate systematically

In terms of gender differences, there were significant associations between previous blood pressure measurement and age, education, income, and working status (self-employed [OR: 0.219, 95% CI: 0.133–0.361], non-working [OR: 0.114, 95% CI: 0.050–0.263], employee [OR:1.000]) among men but not among women. There was a significant association between hypertension and income level (Quartile-2 [OR: 1.984, 95% CI: 1.209–3.255], Quartile-1 [OR: 2.161, 95% CI: 1.415–3.299], Quartile-4 [OR:1]) among women.

Those with a family history of hypertension (OR: 2.019, 95% CI: 1.549–2.633) and those with hypertension (OR: 1.695, 95% CI: 1.280–2.243) also had an increased likelihood of previous blood pressure measurement.

Table 5 shows the multivariable regression analysis for blood pressure measurement and behavioral factors for all participants. Tobacco use (adjusted odds ratio (AOR): 0.538, 95% CI: 0.380–0.761) and salt intake (AOR: 0.247, 95% CI: 0.068–0.893) were associated with a decreased likelihood of previous blood pressure measurement.

**Table 5:** Multivariable logistic regression analysis of previous blood pressure measurement and life-related behavioral factors.

Explanatory variables			AOR	95% CI					*P*-value^1)^
Model-1	Tobacco use	Never use		(		–		)	0.001
		Currently use	**0.538**	(	0.380	–	0.761	)	
Model-2	Alcohol consumption	Never drink		(		–		)	0.994
		Light or moderate drinker	1.010	(	0.728	–	1.400	)	
		Heavy drinker	0.989	(	0.713	–	1.371	)	
Model-3	Fruit and vegetables consumption	Above 5 portions per day		(		–		)	0.676
		Below or 5 portions per day	0.934	(	0.678	–	1.287	)	
Model-4	Physical activity	Above or 150 mins per week		(		–		)	0.625
		Below 150 mins per week	0.923	(	0.670	–	1.273	)	
Model-5	Salt intake	Below 5 g per day		(		–		)	**0.033**
		Above or 5 g per day	**0.247**	(	0.068	–	0.893	)	

Model-1 was adjusted for Age, Education, Family history of hypertension, Gender, Income, Marital status, Working status

Model-2 was adjusted for Education, Family history of hypertension, Gender, Income, Survey language, Working status

Model-3 was adjusted for Education, Family history of hypertension, Income, Marital status, Survey language, Working status

Model-4 was adjusted for Age, Education, Income, Marital status, Survey language, Working status

Model-5 was adjusted for Education, Family history of hypertension, Income, Marital status, Survey language, Working status

^1)^ *P*-value: Bonferroni

## Discussion

### Inadequate blood pressure screening

Participants with normal blood pressure and 26.3% of those who had hypertension had never undergone blood pressure measurement before the STEPS survey. This may suggest that screening for blood pressure was inadequate. Blood pressure measurement is necessary in people with hypertension or people at high risk of hypertension. Early detection and treatment of hypertension can be effective in preventing other diseases [3, 17]. Some of the risk factors for hypertension are preventable, whereas some, such as aging or genetics, are not [9, 17].

A previous qualitative study in Bhutan indicated that there were people who had never undergone blood pressure measurement although they had high blood pressure; however, this previous qualitative study could not show that these results were representative of Bhutan more widely due to the small sample size from a small area [10]. Using a national survey with sample sizes representative of Bhutan [7], we could demonstrate the importance of early detection of hypertension. People with hypertension remain undiagnosed for multiple reasons, including behavioral reasons, lack of awareness, limited access to the health system, patient projections, or medical costs [33–35]. Medical tests should be conducted given their cost-effectiveness. If all the people with no previous blood pressure measurement had normal blood pressure, there would not be a problem. However, this is not the case, so a selective approach is needed to identify people with hypertension or people at high risk for hypertension and which simultaneously does not waste scarce resources.

### Social background factors and gender differences

Married women had more experience with blood pressure measurement than men. For men, aging and working environment were significantly associated with previous blood pressure measurement. However, for women, except for income, there was no significant association between previous blood pressure measurement and working status or aging (Table 4). The socioeconomic factors associated with blood pressure measurement may differ between men and women.

In this study, we could not determine the cause of the gender differences observed in blood pressure measurement; however, existing maternal and child health programs might be the reason that women with a married or cohabiting status had an increased prevalence of previous blood pressure measurement [36, 37]. In Bhutan, maternal and child health services have improved markedly in the past two decades [37, 38]. The government of Bhutan provides free access to basic public health services for all [12]. Nonetheless, among women, those in the above-average income category had a higher coverage of previous blood pressure measurement than those in the below-average income category. This difference might be explained by the travel cost or time cost involved in going to blood pressure measurement appointments [11, 39]. The number of PHCs has increased, and access to health care has improved over the past 30 years [37–40]. An additional 87.7% of the population had access to a PHC within two hours in 2019 [41]. However, traveling for two hours on a mountain road in Bhutan may be difficult for women. Lower-income women might also experience some barriers to visiting the hospital, as previous studies in other countries have indicated, such as insufficient education, gender gap problems, and insufficient family support [42].

We should pay more attention to the low-income group because we need to reduce barriers to accessing health care and promote blood pressure measurement among this group. Previous studies in other countries have also indicated that health-seeking behavior differs by gender [42, 43]. Women have greater awareness and higher consciousness of the treatments for hypertension but simultaneously experienced more barriers to visiting the hospital than men [42–45].

In Bhutan, there are fewer paternal programs for men than there are mother and child health programs; therefore, men might have less chance of having their health screened while healthy. For men, aging is a risk factor for hypertension [46]; therefore, it may be that men tend to go to the hospital after facing health problems as they age. If so, including men in the existing maternal and child health care programs could increase their opportunities to have their blood pressure monitored, encouraging them to pay attention to their own health and the health of their wives, children, and families. Working status had a significant association with the experience of blood pressure measurement among men. Those who are employed may have more opportunities to have their health examined and greater access to other services from their companies than those who are self-employed or non-working [47]. It is possible that housemen or self-employed men must work continuously to obtain a daily income, making it impossible to leave work and seek medical attention, or they may not be able to seek medical attention without other initiatives. Those who are employed may have received some health care through their employer or colleagues. While workplace-based interventions can be effective [45–49], especially among men, methods of providing preventive health management to self-employed, non-working, and low-income people is an issue that remains to be addressed.

### Health behavior and risk perception

People with a family history of hypertension or those with hypertension had more experiences with blood pressure measurement in all analyses most likely because they have an increased awareness of the health threats they might face. People with an unhealthy lifestyle may not be aware of health threats or perceived health vulnerability, and one of the characteristics of people with a healthy lifestyle may be the ability to perceive health threats, regardless of whether they have pre-existing health problems or not. People with an unhealthy lifestyle (high salt intake or tobacco use) had less experience with blood pressure measurement.

Another previous study also showed that risk perception and preventive behavior are closely related [49]. Health perception of hypertension might also be related to health behavior [27, 50].

From the point of view of prevention, it is necessary to promote more blood pressure measurements to those who are at high risk or who have modifiable risk factors. In rural Bhutan, health education and health-related meetings often involve household representatives gathering in PHCs to provide knowledge. This is an effective means of communication due to the characteristics of strong family cohesion in Bhutan [10]. Furthermore, when developing and improving prevention programs, it may be necessary not only to make healthy people healthier but also to approach those at high risk of lifestyle-related diseases effectively and selectively. This selection should occur regardless of whether people are health conscious or not, because 26.3% of subjects in the STEPS survey had never had their blood pressure monitored before.

## Strengths and limitations of this study

This study was rare in that it focused on factors that are related to the experience of blood pressure measurement in Bhutan after adjusting for all possible variables and available confounders. Our study had several strengths. First, the dataset of the “National Survey for Noncommunicable Disease Risk Factors and Mental Health using WHO STEPS Approach in Bhutan– 2014” was representative of the whole of Bhutan, with a 97% valid response rate and the adoption of cluster random sampling methods. Second, the questionnaire included some biomarkers to adjust for risk factors for high blood pressure. Regarding limitations, first, a wish by participants to provide socially desirable answers could have affected the results due to face-to-face interviews. Nonetheless, this was the only method available to collect data from a low literacy rate target population. Second, we cannot deny that there were unmeasurable confounders for some associations that we found in this study, although we tried to adjust for all possible confounders from previous studies.

## Conclusions

To contribute to the early detection of hypertension, we investigated the rate of blood pressure measurement and associated factors in Bhutan. Participants with unhealthy lifestyles had less experience with blood pressure measurement. There was a gender gap in which social determinant factors were associated with previous blood pressure measurement. It is necessary to promote the early detection of hypertension and to pay more attention to low-income women, the self-employed, and non-working men. Levels of blood pressure screening were generally inadequate. Individuals with hypertension or those at high risk for hypertension, such as one-quarter of subjects who had never had their blood pressure monitored before and those who lacked health consciousness, should not be overlooked.

## Supporting information

S1 File Sample size, power calculation, weighting, and adjusting the cluster random sampling procedure. (PDF)

S2 File Directed acyclic graph for multivariable logistic regression. (PDF)
