# Supplementary material for: Social and behavioral factors related to blood pressure measurement: A cross-sectional study in Bhutan
Source: PLoS One. 2022 Aug 17;17(8):e0271914. doi: 10.1371/journal.pone.0271914 (PMC9385017; doi:10.1371/journal.pone.0271914)
Supplement: S1 File — (PDF) [file pone.0271914.s001.pdf]

S1 File. Sample size, power calculation, weighting, and adjusting cluster random sampling procedure

The details of the sampling procedure (Sampling size and power calculation) are mentioned in "National Survey for noncommunicable disease risk factors and mental health using WHO STEPS approach in Bhutan – 2014". In addition, we confirmed weighting procedure details with the Ministry of Health. We have provided these details as supporting information quoted from the report shown below.

Sample size and power calculation

"The Sample size estimate of the number of households to be surveyed with 95% confidence was calculated using the following formula and assumptions.
Where: $n=\{Z2\ 1\text{-}\alpha P(1-P)\}/d2$

Z = level of confidence measure; this represents the number of standard errors away from the mean and describes the uncertainty in the sample mean or prevalence as an estimate of the population mean (normal deviation if alpha equals 0.05, then Z = 1.96 for 95% confidence level). P = baseline level of the indicators. This is the estimated proportion of one of the indicators related to the risk factors currently being measured. The prevalence of overweight and obesity was 52.8% from the last STEPS survey carried out in Thimphu which was the closest value to 50%. d = margin of error. The expected half width of the confidence interval was taken as 0.05 for this study
$n=[1.96*1.96\{0.528(1-0.528)\}]/0.05*0.05$

Four domains were chosen based on men and women and two age groups: younger (18–39 years) and older (40–69 years), providing four age/sex estimates. Taking into account the number of domains and ensuring enough representation by either age-sex groups or urban-rural groups in men and women, and with a design effect of 1.5 to address the issue of cluster sampling, the expected sample size was as follows:
n = 382.9552 * 1.5 * 4 = 2297.7316
Assuming an expected 80% response rate, the final required sample size was 2912.
n = 2297.7316/0.8 = 2872.1646~ (rounded to 2912 for logistical ease)

Out of the targeted 2912 respondents, 2822 (96.9%) participated in STEP I (behavioral measurement), and 2816 (96.7%) participated in STEP 2 (physical measurements). For STEP 3 (biochemical measurement), the response rate for the fasting blood glucose test, excluding non-fasting respondents, was 93.5% (2724 respondents), that for total cholesterol levels was 94.8% (2761 respondents), and urine collection for salt estimation was 89.9% (2618 respondents)."

Weighting procedure:

1. The probability of selection generated during the sampling procedure for SSU and PSU by the Excel sheet provided by the WHO was used.
2. The probability of selection of household level was calculated as shown below:
   = Total number of households sampled in the selected cluster/Total number of households found in the selected cluster during the survey listing
3. Probability of selection of an individual = 1/Household size
4. Population weight:

Age range (in years)	sex	sample		pop		Pop_wt
18–39	1	500	17.7%	148,533	33.4%	1.885408
40–69	1	574	20.3%	87,536	19.7%	0.967893
18–39	2	969	34.3%	133,408	30.0%	0.873797
40–69	2	779	27.6%	75,159	16.9%	0.612345
		2,822		444,636		

5. Non-response rate:
   Since these data were not available stratified by sex and age groups, we calculated them using the location (urban and rural). However, the non-response rate did not differ in these places, and the weight was equivalent to one (see table below). We did not use this weight in the final calculation of the weight.

	Response rate	Non-response weight (1/RR)
rural	0.968253968	1.032786885
urban	0.969866071	1.031070196

6. Final weight calculation
   Individual weight =1/(Probability of selection of PSU * Probability of selection of SSU * Probability of selection of household * Probability of selection of individual)

Final individual weight= individual weight * population weight.

Complex sampling adjustment

We set up complex sampling mode in SPSS (IBM Statistical Package for Social Statistics version 23 with the module for Complex Sample Analysis (IBM Corp., Armonk, NY, USA)), using "Final

individual weight" as mentioned above and "Stratum" ("Rural" and "Urban": 311245 (70%) and 133391(30%)).

The script is as follows:

```
CSPLAN ANALYSIS
  /PLAN FILE='File name'
  /PLANVARS ANALYSISWEIGHT={Final individual weight}
  /PRINT PLAN MATRIX
  /DESIGN STRATA=stratum CLUSTER=psu
  /ESTIMATOR TYPE=EQUAL_WOR
  /POPSIZE MATRIX=stratum;1 311245;2 133391.

CSTABULATE
  /PLAN FILE=File name'
  /TABLES VARIABLES= {each explanatory variable ex) gender marital status} BY {having
experienced blood pressure measurement or not}
  /SUBPOP TABLE= (target population variable) DISPLAY=LAYERED
  /CELLS POPSIZE ROWPCT COLPCT
  /STATISTICS SE CIN(95)
  /MISSING SCOPE=LISTWISE CLASSMISSING=EXCLUDE.
```
