# Supplementary figures and images for: Social and behavioral factors related to blood pressure measurement: A cross-sectional study in Bhutan
Source: PLoS One. 2022 Aug 17;17(8):e0271914. doi: 10.1371/journal.pone.0271914 (PMC9385017; doi:10.1371/journal.pone.0271914)

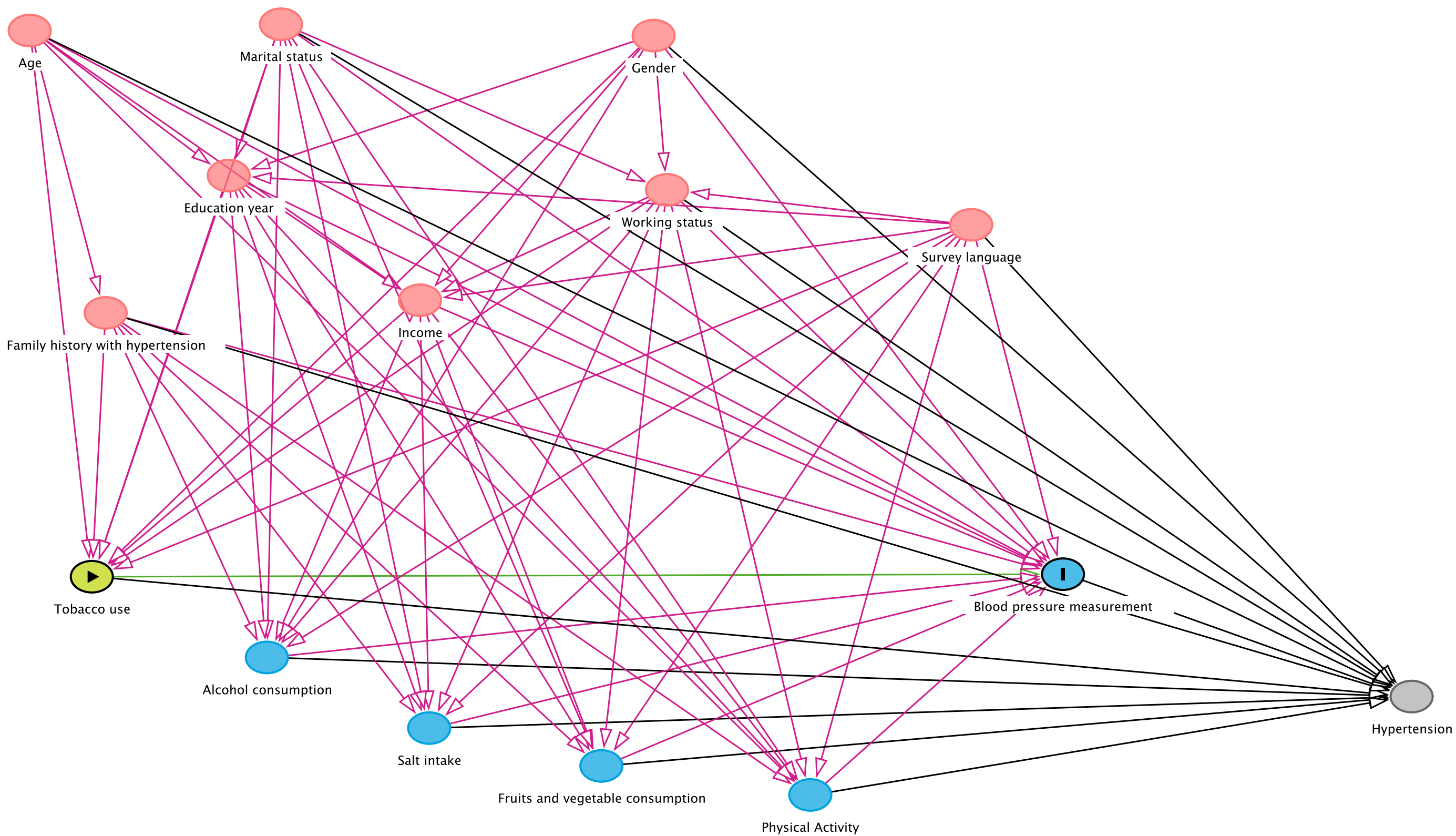

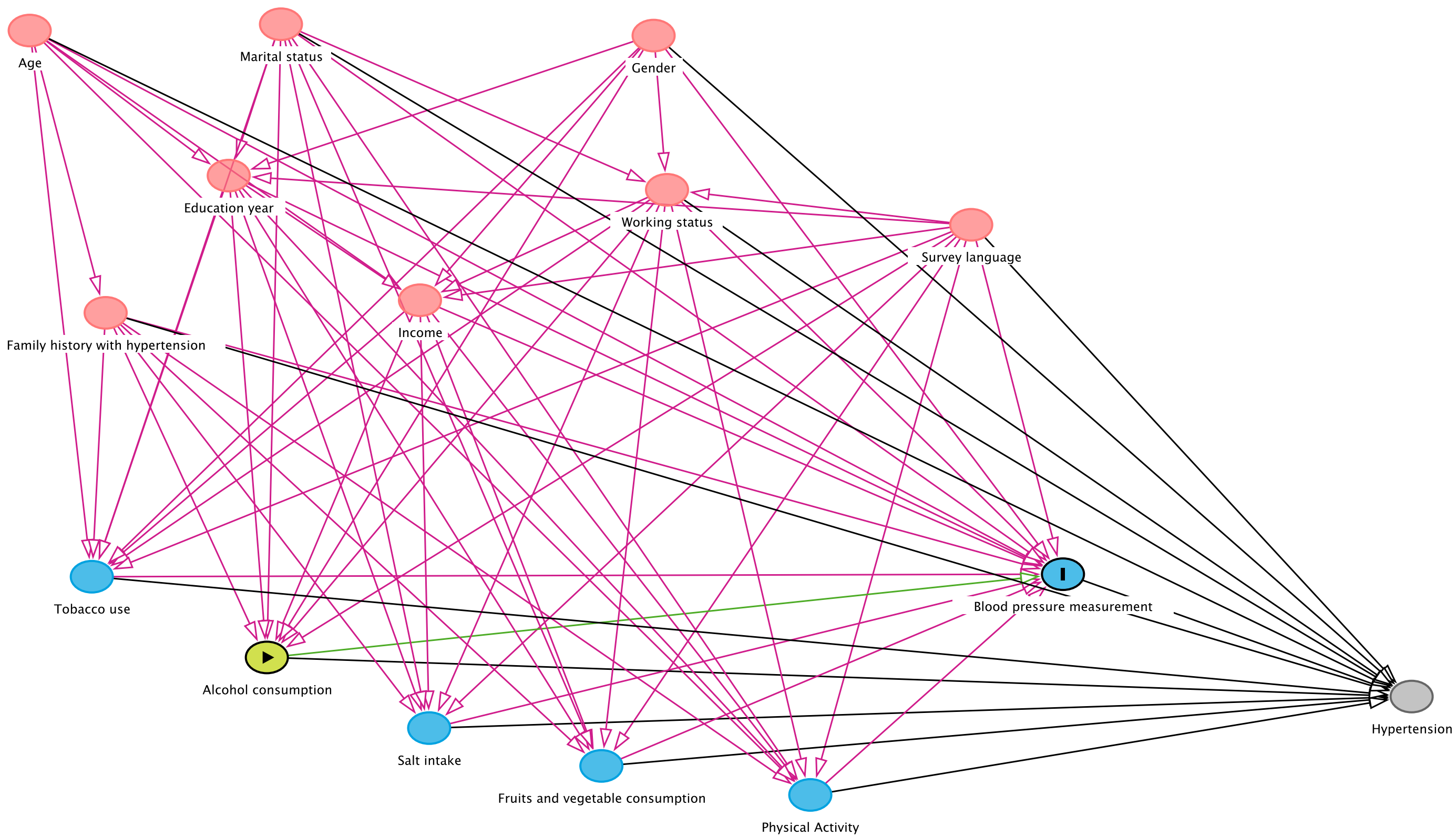

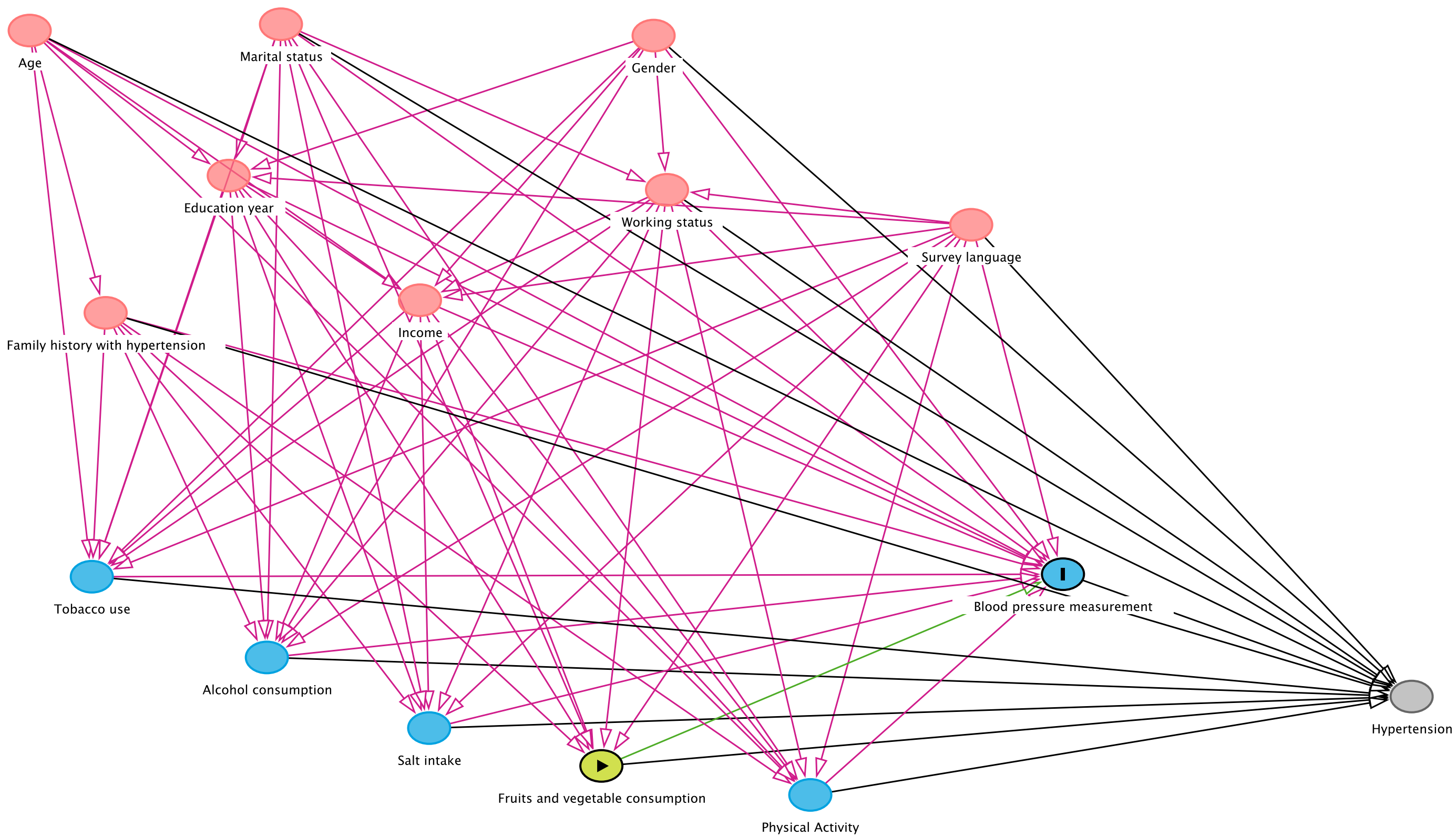

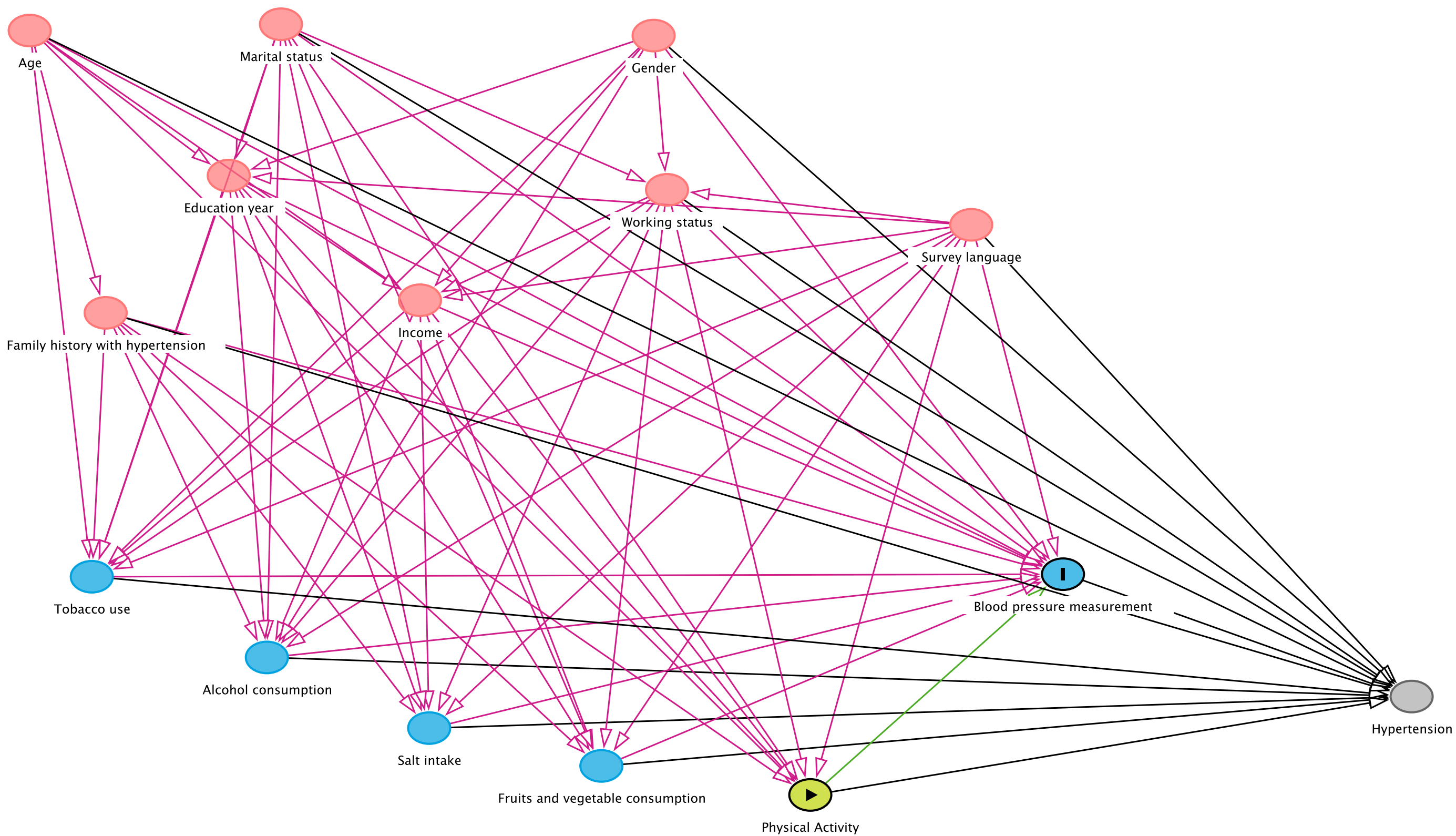

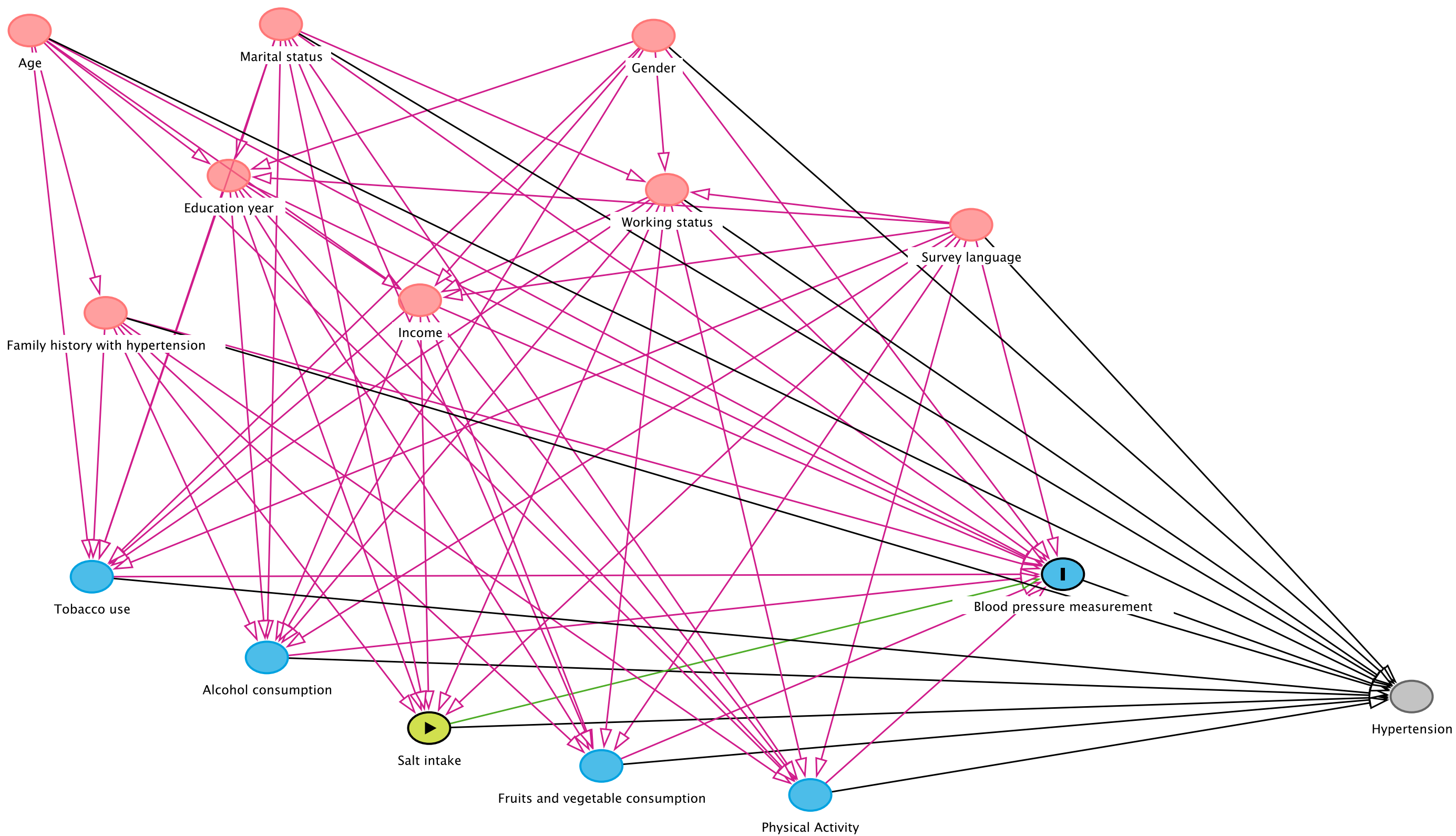

Supplement: S2 File — (PDF) [file pone.0271914.s002.pdf]
