# Supplementary material for: Social and behavioral factors related to blood pressure measurement: A cross-sectional study in Bhutan
Source: PLoS One. 2022 Aug 17;17(8):e0271914. doi: 10.1371/journal.pone.0271914 (PMC9385017; doi:10.1371/journal.pone.0271914)
Supplement: Attachment — Submitted filename: ResponsetoReviewers.docx [file pone.0271914.s003.docx]

Dear Editor and reviewers,

We really appreciate your review and feedback during these difficult times of the COVID-19 pandemic. We have improved our manuscript by responding to your comments, as detailed below:

1. Please ensure that your manuscript meets PLOS ONE's style requirements, including those for file naming. The PLOS ONE style templates can be found at
https://journals.plos.org/plosone/s/file?id=wjVg/PLOSOne_formatting_sample_main_body.pdf and
<https://journals.plos.org/plosone/s/file?id=ba62/PLOSOne_formatting_sample_title_authors_affiliations.pdf>

⇒We have addressed PLOS ONE's style requirements, including those for file naming.

2. Thank you for including your ethics statement:  "The Research Ethics Boards of Health, Ministry of Health of the Royal Government of Bhutan (No. REBH/Approval/2018/089), and the Kyoto University Hospital and Graduate School of Medicine, Ethics Committee (No. R1796) approved the study protocol.".

Please provide additional details regarding participant consent. In the ethics statement in the Methods and online submission information, please ensure that you have specified (1) whether consent was informed and (2) what type you obtained (for instance, written or verbal, and if verbal, how it was documented and witnessed). If your study included minors, state whether you obtained consent from parents or guardians. If the need for consent was waived by the ethics committee, please include this information.

If you are reporting a retrospective study of medical records or archived samples, please ensure that you have discussed whether all data were fully anonymized before you accessed them and/or whether the IRB or ethics committee waived the requirement for informed consent. If patients provided informed written consent to have data from their medical records used in research, please include this information.

Once you have amended this/these statement(s) in the Methods section of the manuscript, please add the same text to the “Ethics Statement” field of the submission form (via “Edit Submission”).

For additional information about PLOS ONE ethical requirements for human subjects research, please refer to <http://journals.plos.org/plosone/s/submission-guidelines#loc-human-subjects-research>.

⇒We have added an explanation of the ethical considerations of our study, as detailed below:

→(p12, line210-215 )　The Research Ethics Boards of Health, Ministry of Health of the Royal Government of Bhutan (No. REBH/Approval/2018/089) and the Ethics Committee of the Kyoto University Hospital and Graduate School of Medicine (No. R1796) approved the study protocol. The above ethics committees waived the requirement for individual informed consent as this secondary analysis used only de-identified data. We have submitted a written pledge of confidentiality to the Ministry of Health of the Royal Government of Bhutan.

3. In your Methods section, please provide additional information about the participant recruitment method and the demographic details of your participants. Please ensure you have provided sufficient details to replicate the analyses such as:

- the recruitment date range (month and year)

⇒Thank you for your comments. The survey “National Survey for Noncommunicable Disease Risk Factors and Mental Health using WHO STEPS Approach in Bhutan – 2014” was conducted from March to June in 2014.

→We added “from March to June”. (p 7, line 127)

- a description of any inclusion/exclusion criteria that were applied to participant recruitment

⇒Thank you for your comments.

In the “National Survey for Noncommunicable Disease Risk Factors and Mental Health using WHO STEPS Approach in Bhutan – 2014”, age 18 to 69 years was an inclusion criterion, and missing data were excluded from the analysis. Furthermore, in this study, pregnant women were excluded from the analysis because of health behavioral and biological differences.

We have rephrased this as shown below:

→(p 11,　lines 202–204)　In the study, individuals aged 18 to 69 years were included. Those with missing data and pregnant women were excluded from the analysis because of health behavioral and biological differences.

- a statement as to whether your sample can be considered representative of a larger population

⇒Thank you for your comments.

We have added the following phrase:

→(p 11, line 198-197)　 “to ensure they were representative of the national population”

- a description of how participants were recruited

- descriptions of where participants were recruited and where the research took place."

⇒Thank you for your comments. We have added the following sentences:

→(p 8, lines 134–135)　“According to the sampling plan, trained health staff recruited participants in each sampling field where the participants were living.”

Further information about survey procedure can be obtained from the report “National Survey for Noncommunicable Disease Risk Factors and Mental Health Using WHO STEPS Approach in Bhutan – 2014”. We have provided supporting information quoted from the report (S1 File).

4. Please provide a sample size and power calculation in the Methods, or discuss the reasons for not performing one before study initiation.

⇒Thank you for your comments. The survey “National Survey for Noncommunicable Disease Risk Factors and Mental Health Using WHO STEPS Approach in Bhutan – 2014” already considered the sample size and power calculation. Hence, we did not mention it in this paper; however, additional information from the Method part quoted from the report (S1 file) is now provided as supporting information.

Sample size and power calculation

“The Sample size estimate of the number of households to be surveyed with 95% confidence was calculated. The prevalence of overweight and obesity was 52.8% from the last STEPS survey carried out in Thimphu which was the closest value to 50%. d = margin of error. The expected half width of the confidence interval was taken as 0.05 for this study

　N=[1.96*1.96{0.528（1-0.528）}]/0.05*0.05

Four domains were chosen based on men and women and two age groups: younger (18–39 years) and older (40–69 years), providing four age/sex estimates. Taking into account the number of domains and ensuring enough representation by either age-sex groups or urban-rural groups in men and women, and with a design effect of 1.5 to address the issue of cluster sampling, the expected sample size was as follows:

n = 382.9552 * 1.5 * 4 = 2297.7316

Assuming an expected 80% response rate, the final required sample size was 2912.

n = 2297.7316/0.8 = 2872.1646~ (rounded to 2912 for logistical ease)

5. We note that the grant information you provided in the ‘Funding Information’ and ‘Financial Disclosure’ sections do not match.

When you resubmit, please ensure that you provide the correct grant numbers for the awards you received for your study in the ‘Funding Information’ section.

⇒Thanks so much for your point out. We have addressed.

→(p26,　Line438-449)　Our research was supported by

1)	Hiromi Kohori Segawa, K. MATSUSHITA FOUNDATION, Grant Number 19-048 http://matsushita-konosuke-zaidan.or.jp/en/

2)	Yuichi Imanaka, Program of Collaborative Research at the Centre for Southeast Asian Studies (IPCR-CSEAS), Kyoto University 2018 type4 https://ipcr.cseas.kyoto-u.ac.jp/

3)	Hiromi Kohori Segawa, Program of Collaborative Research at the Centre for Southeast Asian Studies (IPCR-CSEAS), Kyoto University 2020 type7 https://ipcr.cseas.kyoto-u.ac.jp/

4)	Yuichi Imanaka, JSPS KAKENHI Grant Number JP19H01075 from the Japan Society for the Promotion of Science https://www.jsps.go.jp/english/

The funders played no role in the study design, data collection and analysis, decision to publish, or preparation of the manuscript.

6. Thank you for stating the following in your Competing Interests section:

“NO”

Please complete your Competing Interests on the online submission form to state any Competing Interests. If you have no competing interests, please state "The authors have declared that no competing interests exist.", as detailed online in our guide for authors at http://journals.plos.org/plosone/s/submit-now

 This information should be included in your cover letter; we will change the online submission form on your behalf.

⇒Thank you for your comments. We have mentioned this in our cover letter with the following information.

→The authors have declared that no competing interests exist. The funders played no role in the study design, data collection and analysis, decision to publish, or preparation of the manuscript.

7. We note that you have indicated that data from this study are available upon request. PLOS only allows data to be available upon request if there are legal or ethical restrictions on sharing data publicly. For more information on unacceptable data access restrictions, please see http://journals.plos.org/plosone/s/data-availability#loc-unacceptable-data-access-restrictions.
In your revised cover letter, please address the following prompts:
a) If there are ethical or legal restrictions on sharing a de-identified data set, please explain them in detail (e.g., data contain potentially sensitive information, data are owned by a third-party organization, etc.) and who has imposed them (e.g., an ethics committee). Please also provide contact information for a data access committee, ethics committee, or other institutional body to which data requests may be sent.

b) If there are no restrictions, please upload the minimal anonymized data set necessary to replicate your study findings as either Supporting Information files or to a stable, public repository and provide us with the relevant URLs, DOIs, or accession numbers. For a list of acceptable repositories, please see http://journals.plos.org/plosone/s/data-availability#loc-recommended-repositories.

We will update your Data Availability statement on your behalf to reflect the information you provide.

⇒Thank you for your comments. We have rephrased this information as shown below.

→(p 26,　lines 429–436)　Since the Ministry of Health, Bhutan is the authority for the data, the authors were not able to make it available in this manuscript. However, the original report (World Health Organization and Ministry of Health in Bhutan. National Survey for Noncommunicable Disease Risk Factors and Mental Health using WHO STEPS Approach in Bhutan – 2014) is available at https://apps.who.int/iris/handle/10665/204659. Requests to access the raw data can be directed to the Research Ethics Board of Health, Ministry of Health, Bhutan, or the Royal Government of Bhutan.

Additional Editor Comments (if provided):
[Note: HTML markup is below. Please do not edit.]
Reviewers' comments:
Reviewer's Responses to Questions

**Comments to the Author**
1. Is the manuscript technically sound, and do the data support the conclusions?

The manuscript must describe a technically sound piece of scientific research with data that supports the conclusions. Experiments must have been conducted rigorously, with appropriate controls, replication, and sample sizes. The conclusions must be drawn appropriately based on the data presented.

Reviewer #1: No　　　　　　Reviewer #2: Yes　　Reviewer #3: Partly

 2. Has the statistical analysis been performed appropriately and rigorously?

 Reviewer #1: No　　Reviewer #2: Yes　　Reviewer #3: I Don't Know

 3. Have the authors made all data underlying the findings in their manuscript fully available?

The [PLOS Data policy](http://www.plosone.org/static/policies.action#sharing) requires authors to make all data underlying the findings described in their manuscript fully available without restriction, with rare exception (please refer to the Data Availability Statement in the manuscript PDF file). The data should be provided as part of the manuscript or its supporting information, or deposited to a public repository. For example, in addition to summary statistics, the data points behind means, medians and variance measures should be available. If there are restrictions on publicly sharing data—e.g. participant privacy or use of data from a third party—those must be specified.

 Reviewer #1: No　　Reviewer #2: No　　Reviewer #3: Yes

 4. Is the manuscript presented in an intelligible fashion and written in standard English?
PLOS ONE does not copyedit accepted manuscripts, so the language in submitted articles must be clear, correct, and unambiguous. Any typographical or grammatical errors should be corrected at revision, so please note any specific errors here.

 Reviewer #1: No　　Reviewer #2: Yes　　Reviewer #3: Yes

 5. Review Comments to the Author
Please use the space provided to explain your answers to the questions above. You may also include additional comments for the author, including concerns about dual publication, research ethics, or publication ethics. (Please upload your review as an attachment if it exceeds 20,000 characters)

 Reviewer #1: The major problems is the methodology and analysis done did not fit the study objectives. The study objective is to determine the factors associated with blood pressure measurement. It is not clear in the method the operational definition of blood pressure measurement in this study. What is the main outcome in this study? Is it behaviour practice of BP measurement for the past? duration or ever checked BP?

⇒Thank you for these important comments. We acknowledge the limitation of our explanation. We would like to make it clearer.

During the survey “National Survey for Noncommunicable Disease Risk Factors and Mental Health using WHO STEPS Approach in Bhutan – 2014”, blood pressure was checked 3 times by health workers. We could identify the gap between self-reported hypertension and examination based on the survey time (including diagnosis by health staff before the survey or current medication treatment). We believe that this gap is critical because individuals who have hypertension but are unaware should start to control their blood pressure. In particular, those who answered that they had no experience with blood pressure monitoring should be given attention. Periodic blood pressure monitoring is very important. As public health workers prevent worsening hypertension, we need to pay adequate attention to those with hypertension who did not have their blood pressure monitored before the survey. Hence, we wanted to identify what factors prevented them from being aware of their hypertension status before the survey. We wanted to identify whether they had concerns about their health but were unable have their blood pressure monitored or were unaware of their risk.

In the current study, we first wanted to identify factors related to experience with blood pressure monitoring before the survey. Second, we wanted to explore what factors are related to experience with blood pressure measurement among individuals with hypertension before the survey before the survey.

To make these factors clear, we have made the following changes:

1) We have added second objective “To explore what factors are related to experience with blood pressure measurement among individuals with hypertension before the survey” (p 6,　lines 110–111 )
2) We have added information about the time of diagnosis of hypertension (during or before the survey).
3) We have improved the explanation of the definition of hypertension used in this study.

→(p 9-10,　line 167-170 )　 Hypertension was defined by the following criteria: (1) systolic blood pressure (SBP) ≥ 140 mmHg or diastolic blood pressure (DBP) ≥ 90 mmHg measured as the average of three measurements taken by health staff in the survey; (2) a previous diagnosis of hypertension by healthcare workers; (3) current treatment with medication for hypertension

Reviewer #2: Reviewer Comments
Kohori-Segawa et al conducted a study to determine what characteristics are associated in Bhutanese people with the experience of having a blood pressure measurement. I find the article very interesting and neatly conducting. I have a few questions regarding the design:
1) In line x you mention patients with hypertension that had not had blood pressure measured, can you specify then how was the diagnosis made? Do you have any information regarding this? You mention this again in the conclusions in which you say people with hypertension have never had blood pressure measurements in their life so I do not understand how they know their hypertension status. Is it possible that they do not remember having their blood pressure measured rather than the measurement not being performed?

⇒Thank you for your comments. We acknowledge the shortcomings in the explanation. We would like to clarify. (Your comments are similar to the comments from reviewer 1; therefore, our responses are repeated.)

During the survey “National Survey for Noncommunicable Disease Risk Factors and Mental Health using WHO STEPS Approach in Bhutan – 2014”, blood pressure was checked 3 times by health workers. We could identify the gap between self-reported hypertension and examination based on the survey time (including diagnosis by health staff before the survey or current medication treatment). We believe that this gap is critical because individuals who have hypertension but are unaware should start to control their blood pressure. In particular, those who answered that they had no experience with blood pressure measurement should be given attention. Periodic blood pressure monitoring is very important. As public health workers prevent worsening hypertension, we need to pay adequate attention to those with hypertension who did not have their blood pressure measured before the survey. We wanted to identify whether they had concerns about their health but were unable have their blood pressure measured or were unaware of their risk.

To make these factors clear, we have made the following changes:

1) We have added second objective “To explore what factors are related to experience with blood pressure measurement among individuals with hypertension before the survey” (p 6,　lines 110–111 )
2) We have added information about the time of diagnosis of hypertension (during or before the survey).
3) We have improved the explanation of the definition of hypertension used in this study.

→(p 9-10,　line 167-170 )　 Hypertension was defined by the following criteria: (1) systolic blood pressure (SBP) ≥ 140 mmHg or diastolic blood pressure (DBP) ≥ 90 mmHg measured as the average of three measurements taken by health staff in the survey; (2) a previous diagnosis of hypertension by healthcare workers; (3) current treatment with medication for hypertension

There is a possibility that the participants might not remember having their blood pressure measured as you mentioned. Such an unconscious measurement can be counted as not measured. If the participants forgot that they had their blood pressure measured, this indicates that they were not aware of the blood pressure measurement, so it is not a problem to count it as not having been taken. The important point is finding the gap in their practices, their wishes, and their real risk.

2) Regarding multivariable analysis, I would like for you to specify adjustment variables rather than just listing the variable category (line 162)

⇒Thank you for your comments. We specified this point in the explanatory variables’ subsection (adjustment variables). To clarify, we added the explanation shown below and deleted the categories.

→(p 10,　line 177 )　 as mentioned in the explanatory variables.

3) In line 317 you mention than unemployed or self-employed men are less likely to have their blood pressure measured. A possible explanation could be the need to work on a daily basis to obtain income which makes it impossible to leave and seek for medical attention. I would like to know your thoughts about this.
⇒We acknowledge your valuable comments.

We have added the following explanation:

→(p 22–23,　lines 366–370 )　 It is possible that non-working or self-employed men must work on a daily basis to obtain income, making it impossible to leave work and seek medical attention, or they may not be able to seek medical attention without other interactions. Those who are employed may have received some health care through their employer or colleagues.

Finally I would suggest changing the wording in sentences mentioning previous studies.

⇒Thank you for your comments.　We have changed the wording about previous studies.

(p 20,　line 323 )　previous study →　A previous qualitative study

(p 20,　lines 324–325 )　this study →　this previous qualitative study

Reviewer #3:

1- Line 184: Can't find Figure1?. Does it refer to Table1?

⇒Thank you for your comments. It was Figure 1. PlosOne indicated to display the figures in different files not in the manuscript. Hence, we followed these directions.

2- It is difficult to understand the % in last column in table 1. How these % were calculated?

⇒Thank you for your comments. We calculated these data with the weight adjusted and adjusted for multistage cluster random sampling with SPSS. The total percentage is not 100 percent due to this adjustment. The last column is the point estimates after the adjustment. We did not include confidence intervals, which might have been confusing. Therefore, we have added 95% CIs and mentioned “estimates” instead of “adjusted” in Table 1. Furthermore, we have improved the footnotes in Table 1 and added S1 File.

(p 14 )

1) Weight adjusted % (S1 File)

2) Population with blood pressure monitoring using the questionnaire item “Have you ever had your blood pressure measured by a doctor or other health worker?” Answers of “yes” were counted.

3) Estimated % adjusted with complex sampling: Population with blood pressure monitoring/Total target population (S1 File)

We have added the following explanation in the supporting information (S1 File).	.

Weighting procedure:

1. The probability of selection generated during the sampling procedure for SSU and PSU by the Excel sheet provided by the WHO was used.
2. The probability of selection of household level was calculated as shown below:

= Total number of households sampled in the selected cluster/Total number of households found in the selected cluster during the survey listing

3. Probability of selection of an individual = 1/Household size
4. Population weight:

| Age range (in years) | sex | sample |  | pop |  | Pop_wt |
| --- | --- | --- | --- | --- | --- | --- |
| 18–39 | 1 | 500 | 17.7% | 148,533 | 33.4% | 1.885408 |
| 40–69 | 1 | 574 | 20.3% | 87,536 | 19.7% | 0.967893 |
| 18–39 | 2 | 969 | 34.3% | 133,408 | 30.0% | 0.873797 |
| 40–69 | 2 | 779 | 27.6% | 75,159 | 16.9% | 0.612345 |
|  |  | 2,822 |  | 444,636 |  |  |

5. Non-response rate:

Since these data were not available stratified by sex and age groups, we calculated them using the location (urban and rural). However, the non-response rate did not differ in these places, and the weight was equivalent to one (see table below). We did not use this weight in the final calculation of the weight.

[Reference] World Health Organization. WHO STEPS Surveillance Manual. 2017. Available from https://www.who.int/ncds/surveillance/steps/STEPS_Manual.pdf

Individual weight =1/(Probability of selection of PSU * Probability of selection of SSU * Probability of selection of household * Probability of selection of individual)

Final individual weight= individual weight * population weight.

We set up complex sampling mode in SPSS (IBM Statistical Package for Social Statistics version 23 with the module for Complex Sample Analysis (IBM Corp., Armonk, NY, USA)), using “Final individual weight” as mentioned above and “Stratum” (“Rural” and “Urban”: 311245 (70%) and 133391(30%)).

The script is as follows:

CSPLAN ANALYSIS

/PLAN FILE='File name'

/PLANVARS ANALYSISWEIGHT={Final individual weight}

/PRINT PLAN MATRIX

/DESIGN STRATA=stratum CLUSTER=psu

/ESTIMATOR TYPE=EQUAL_WOR

/POPSIZE MATRIX=stratum;1 311245;2 133391.

CSTABULATE

/PLAN FILE=File name'

/TABLES VARIABLES= {each explanatory variable ex) gender marital status} BY {having experienced blood pressure measurement or not}

/SUBPOP TABLE= (target population variable) DISPLAY=LAYERED

/CELLS POPSIZE ROWPCT COLPCT

/STATISTICS SE CIN(95)

/MISSING SCOPE=LISTWISE CLASSMISSING=EXCLUDE.

3-How to identify potential confounders? What are the potential confounders' variables to be included in multivariate analysis?

⇒We acknowledge your valuable comments. Health behavior such as blood pressure measurement is known to be affected by various factors. All of these various factors are associated with each other.

Kenneth et al (1988) proposed an ecological model for health promotion that focuses attention on both individual and social environmental factors as targets for health promotion interventions. It is important that “interpersonal” processes shape human behavior [1].

Barton H et al (2006) also displayed the determinants of health and wellbeing in a human habitation map and mentioned “lifestyle” in various factors [2].

Reflecting on the Bhutanese situation, health policy is common for all Bhutanese across the country. Some but not all of the primary prevention programs for NCDs, such as displays of posters in health facilities, had already started in 2014 at the time of the survey. The number of primary health centers (PHCs) was also increasing in rural areas due to expansion of primary health care. Thus, we predicted that the situations of diagnosis and risk perception were also diverse. Furthermore, risk perception (threat) is also known to affect human behavior [3].

We selected individual and social environmental factors affecting interpersonal decisions or close networks, lifestyle-related factors, health conditions of hypertension, and family history of hypertension as biological risk perception factors.

1. McLeroy KR, Bibeau D, Steckler A, Glanz K. An Ecological Perspective on Health Promotion Programs. Health Education Quarterly. 1988;15(4):351-77.
2. Barton H, Grant M. A health map for the local human habitat. Journal of the Royal Society for the Promotion of Health. 2006;126(6):252-3.
3. Rosenstock IM, Strecher VJ, Becker MH. Social learning theory and the Health Belief Model. Health Educ Q. 1988;15(2):175-83.

According to the abovementioned theories, we selected the variables from “National survey for noncommunicable disease risk factors and mental health using WHO STEPS approach in Bhutan – 2014”.

We selected that gender and marital status; age, level of education, working status, residential area (urbanicity), income, and survey language Tobacco use, alcohol consumption, fruits and vegetables consumption, physical activity, estimated salt intake, having hypertension, and family history of hypertension. We put all these variables we selected to our models forcefully, after we checked the interaction effect and coefficient, excluding the residential area (urbanicity) due to adjusting cluster random sampling.

We improved our explanation on the statistical methods and deleted the categories in explanatory

4-I am not English native speaker, but I just feel that there might be a need to further improvement in English writing fashion?

⇒Thank you for your comments. English is our second language. We have asked a professional English copyeditor to review and edit our manuscript to ensure that it is written in standard English.

5-Title: It is too long; it should be revised?

⇒Thank you for your comments. We have shortened our title as below.

Old Title: Factors related to blood pressure measurement, with attention needed for unemployed men, poor women, and people’s health behaviors: A cross-sectional study in Bhutan

Revised title: Social and behavioral factors related to blood pressure measurement: A cross-sectional study in Bhutan

6-Income: Readers who are not from the Kingdom of Bhutan may not understand like "Nu.0–9,000; ...." Is it possible to convert into a commonly known like UDS?

⇒We acknowledge your valuable comments. We converted from Bhutanese Ngultrum (Nu) into USD.

→(p 9,　lines 145–147 )

1 USD = 74.4707 BTN (Accessed on 11^th^ August 2021 https://www.xe.com/)

Nu. 0–9,000　→0–120.8 USD

Nu. 9,001–30,000　　→120.9–402.9 USD

Nu. 30,001–60,000　　→403.0–805.7 USD

Nu. 60,001+　　→805.8+ USD

7-Discussion: Authors raised about "Considering barriers......" (line 284-285) and "Contradictions in health behavior...." (line 320), these are all the factors associated with blood pressure measure. The words .... barriers, contradictions might not appropriate to be used here?

⇒Thank you for your comments. We reconsidered appropriate subtitles for the discussion. We agree with your suggestion that the barriers or contradictions should be discussed in phase but are not needed in the title. We have now changed the subtitles in the discussion.

→(p 21,　line 334)　Considering barriers to self-employed or unemployed men, and poor women →Self-employed or non-working men and low income women

→(p 23,　line 373)　Contradictions in health behavior and risk perception → Health behavior and risk perception

 6. PLOS authors have the option to publish the peer review history of their article ([what does this mean?](https://journals.plos.org/plosone/s/editorial-and-peer-review-process#loc-peer-review-history)). If published, this will include your full peer review and any attached files.
If you choose “no”, your identity will remain anonymous but your review may still be made public.
**Do you want your identity to be public for this peer review?** For information about this choice, including consent withdrawal, please see our [Privacy Policy](https://www.plos.org/privacy-policy).

 Reviewer #1: No　Reviewer #2: **Yes:** Mercedes Aguilar-Soto　　Reviewer #3: No　Author：Yes
