# Supplementary material for: Social and behavioral factors related to blood pressure measurement: A cross-sectional study in Bhutan
Source: PLoS One. 2022 Aug 17;17(8):e0271914. doi: 10.1371/journal.pone.0271914 (PMC9385017; doi:10.1371/journal.pone.0271914)
Supplement: Attachment — Submitted filename: Responsetoreviewers.docx [file pone.0271914.s004.docx]

Dear Editor and reviewers,

We really appreciate your review and feedback during these difficult times of the COVID-19 pandemic. We took long time to learn causal inference and modify. However, now we have improved our manuscript by responding to your comments, as detailed below:

Reviewer #1: The author has adequately addressed all comments-based form previous reviewers and the manuscript seem to be sound. However, I would like to advise the author to add the p value for the multivariate or bivariate analysis and highlight- bold the results that are significant. This can help the readers to recognized those significant factors in the table easily.

⇒Thank you so much for your advice. I added p-value as much as possible and made them bold for significant results. However, in complex-sampling module Ver23, we are not able to calculate p-value individually in our logistic multivariable regression model. I asked IBM company, they guided us if we want to get p-value individually we need to change all references in our data set.

Reviewer #2: Yuichi Imanaka et al conducted a study to determine social and behavioral factors that determine having blood pressure measurements in Bhutan. They have corrected prior comments but there are some changes that are still needed.

Line 55: had a lower likelihood of undergoing blood pressure measurement
Correct wording.
⇒Thank you for your comments. However, we think the wording are correct. We think that likelihood Is better than odds in this case due to complex survey module, and proofed by professional English editor. In case of “undergoing “makes confuse, we changed the word from” undergoing” to “having experience”. (Line59, 62)

I would further specify everytime you mention hypertensive patients did not have their blood pressure measured that this was after the first diagnosis measurement. (Line 65, 66)
⇒Thank you for your comments. We added “before the STEPS survey” and modified explanation. (Line 67-68, 96,104-106, 114-116, 173,289,365)

LINE 88 : Have showed to be necessary instead of has
⇒Thank you for your advice.　We have changed from “have” to “has”.(Line90)

Again in line 94 you mention people with hypertension have not had their blood pressure measured but this is impossible. Please address this like you did previously. In line 96 you mention according to NCD survey reports, “Around 96 (31.3%) of the study population had never had their blood pressure checkup”, but this again does not make sense if they already have the diagnosis. They needed at least 2 measurements performed priorly. Perhaps you should clarify “follow-up” blood pressure in all sentences regarding new measurements.
⇒Thank you for your comments. I added explanation “before the previous field work conducted in 2017”. However, we can’t modify the quoted sentences from previous government NCD survey report. We mention whom have no experience of high blood measurement before we met or before survey. During field work or the survey, we diagnosed their hypertension. (Line96)

Line 163: How was salt intake estimated? Based on a diet questionnaire?
⇒Thank you for your comments. We estimated salt intake based on spot urine. Details of the method to estimate salt intake are now included in the manuscript.

Salt intake was calculated using the Tanaka Formula (Tanaka T, Okamura T, Miura K, Kadowaki T, Ueshima H, Nakagawa H, et al. A simple method to estimate populational 24-h urinary sodium and potassium excretion using a casual urine specimen. Journal of Human Hypertension. 2002;16(2):97-103).

Salt Intake per day(g) = (21.98 * ((Spot Urinary Sodium/

(Spot Urinary Creatinine *10/0.0884)) * (((14.89* Weight (Kg))+(16.14*height(m))*(2.04*age))-2244.45))**0.392)/17.1 (Line167-169)

Line 170: I do not understand why you used family history on hypertension as a definition for diagnosis. I suggest you remove this definition.
⇒Thank you for your suggestion. We have removed it. (Line175-176)

Line 176: I do not understand what you mean by this: All of the data are available and considered to be related to the dependent variable from the previous literature as mentioned in the explanatory variables.
⇒Thank you for your comments. We have improved the sentence as below

All available and selected data from the STEPS survey are considered related to the objective variable from previous literature. Among those variables that may be related to blood pressure measurement, we selected the explanatory variables to be fed into the model using the Directed Acyclic Graph (S3 File). (Line180-183)

I would suggest you specify very clearly which patients were included: only patients who had prior diagnosis of hypertension defined as known diagnosis or those who were diagnosed during the National Survey for Noncommunicable Disease Risk Factors and Mental Health.
⇒Thank you for your suggestion. Our explanation may make you confuse. We include all aged 18 to 69 years. We didn’t exclude normal blood pressure(Figure1). We just evaluate the hypertension during NCD STEPS survey. We added the explanation of the definition about hypertension through our paper.

Line 205: Again, it is not clear why you made sub-analysis for people with hypertension, you need to be very careful with wording as to say that your objective is to know factors that determine the experiences of having blood pressure measurements and whether they differ for patients who had prior hypertension diagnosis and those who did not.
⇒Thank you for your meaningful advice.　We deeply considered and erased the subgroup analysis for multivariable regression models.”

In the results section, if variables have a parametric distribution, it is better to state mean and standard deviation rather than standard error.
⇒Thank you for your advice. We have added standard deviations in Table1. We recognize that logistic regression does not matter whether variables have a parametric distribution or not. And we adjusted the weight and cluster using SPSS complex sampling module, so it is better to use Standard Error than Standard Deviation. So, we kept standard error too. (Table1,Line,206-207,231)

In table 1 rename variable Gender_Marital Status since it looks like you just left the statistical program output without correcting variable names.
⇒Thank you for your advice. We deeply considered and changed not only names but also our models. We divided the combined variables as original. Because we can show the effects individually. (Line153-154, S3File)

In table two remove the parenthesis after 1 and 2 super indexes. Income variable in table 2 is not in the same format as the rest of the table.
⇒Thank you for your advice. We improved it.

In line 317 you still insist that patients with hypertension never underwent blood pressure measurement in their lives, but this, again, is not possible since that is the only way to diagnose hypertension is measuring blood pressure. You have explained in previous responses what you mean but you need to be very explicit about it throughout your discussion.
⇒Thank you for your meaningful advice.

We have added the explanation throughout our discussion. All descriptions include the definition of Hypertension in this paper so that reader can understand who has never had a blood pressure measurement prior to the NCD STEPS survey. Hypertension was determined during the NCD STEPS survey, and the experience of blood pressure measurement was prior to the NCD STEPS survey. (Line 67-68, 96,104-106, 114-116, 173,289,365)

From this your advice, we also learned about causal inferences in the field of epidemiology, considered that there is a clear time difference between the blood pressure measurement experience and the hypertension, and reconsidered our multivariate analysis model. The variable of hypertension should be collider in our models (S3 File).

Check wording in line 352.

⇒Thank you for advice. We have changed as below

Lower income women might also experience some barriers to visit the hospital, as previous studies in other countries have indicate. (Line324-325)

Reviewer #3: I have no idea whether data analysis was done appropriately and rigorously. However, the presentation of the results is clear and response to the research objectives.

⇒Thank you so much for your comments. Regarding the data analysis, we have done the previous revisions rigorously. However, we have reconsidered and changed models from view point of causal inference. There was no significant change in the results from the last revision.
