# Supplementary material for: Social and behavioral factors related to blood pressure measurement: A cross-sectional study in Bhutan
Source: PLoS One. 2022 Aug 17;17(8):e0271914. doi: 10.1371/journal.pone.0271914 (PMC9385017; doi:10.1371/journal.pone.0271914)
Supplement: Attachment — Submitted filename: Response to reviewers.docx [file pone.0271914.s005.docx]

May 20 2022

Editor-in-Chief 　PLOS ONE

Dear Dr. Marcelo Arruda Nakazone,

We appreciate your review and feedback. Please find enclosed our revised manuscript entitled “Social and behavioral factors related to blood pressure measurement: A cross-sectional study in Bhutan”

We have addressed all of the reviewers’ comments as below

1) In line 174 the final definition of hypertension is having medication for hypertension and a positive family history of hypertension. I would suggest citing where was this definition taken from or how do you justify it.

⇒Thank you for your suggestion. We realized that the definition was mistakenly written as “and a positive family history of hypertension”. Therefore, we removed it.

2) Line 199 repeats again in line 201.

⇒Thank you for your confirmation. Starting words were the same as “we evaluated…”. Actually, there is not repetition in the contents. We changed the sentence to better the understanding.

Line 199: We evaluated the association between the experiences of having blood pressure measurements and individual and social environmental factors affecting interpersonal decisions or close networks through descriptive and univariable logistic regression, the association between the experiences of having blood pressure measurements and high-risk behaviors of hypertension factors through descriptive and multivariable logistic regression.

3) In line 203 I would further clarify which variables you identified as confounders in your multivariable analyses.

⇒Thank you for your advice. We added the explanation as below.

Line203-206: Through Directed Acyclic Graph, we defined minimal sufficient adjustment sets for estimating the total effect of high-risk behaviors of hypertension factors on the experiences of having blood pressure measurements.

4) Line 212-216 are written incorrectly.

⇒Thank you reviewer. We modified the sentence as below.

Line214: In the study, participants aged 18 to 69 years were included. Variables with missing data and pregnant women were excluded from the analysis because of health behavioral and biological differences.

5) Line 259 is missing reference.

⇒Thank you reviewer for this notification.

Actually, the reference here relates to reference of Odds calculated (i.e. no formal education). We changed from (Ref) to (OR:1).

6) In line 266 where you say you did not find an association between Q3 and the experience of blood pressure measurement, why are you focusing only on Q3? If it is because it is the only one with a statistical association you should write it like that. And why are highest income quartiles categorized as Q1? Usually Q1 is the lowest quartile.

⇒Thank you reviewer for this critical comment and the valuable suggestion. We modified the sentence as below.

Line263: In terms of income, all participants in the Q4 (Nu. 60,001+: 805.8+ USD (OR:2.404, 95% CI:1.575 –3.667)) and Q3 category (Nu 30,001–60,000: 403.0–805.7 USD (OR:2.546, 95% CI: 1.640–3.955)) had an increased likelihood of having experienced blood pressure measurement compared with those in the lowest income category Q1 (Nu 0–9,000: 0–120.8 USD[OR:1]). We could find the significant association between Q1, Q3, Q4 and the experience of blood pressure measurement, but we could not find a significant association between Q2 (Nu 9,001–30,000: 120.9–402.9 USD) and the experience of blood pressure measurement.

7) Line 292 is repetitive using diseases twice.

⇒ “cardiovascular diseases” is removed from the sentence

Line 294: Early detection and treatment of hypertension can be effective in preventing other diseases [3,17]

8) Line 306 background should be written as one word.

⇒Thank you. “Back ground” is changed to single word “background” as suggested

9) Line 306 this subtitle does not accurately reflect what you explain in the paragraph.

⇒Thank you for this critique. We modified from “Social background factors and gender difference: Self-employed, non-working or low-income men, and low-income women” to “Social background factors and gender differences”.

10) In line 324 you mention other barriers but not explain further which ones.

We added the explanation as below.

Line326-328: Lower income women might also experience some barriers to visiting the hospital, as previous studies in other countries have indicated, such as insufficient education, gender problems, insufficient family supports, etc. [42].

11) Line 338 should say “have more opportunities on having their health examined” rather than the actual wording.

⇒Thank you reviewer for your suggestion. We modified the sentence as below.

Line341: Those who are employed may have more opportunities on having their health examined and other services from their companies than those who are self-employed or non-working [47].

12) In line 347 I would further explain your hypotheses on this association or move line 352 next to this paragraph.

⇒We moved the sentences as suggested.

13) Line 350 is repetitive, I would suggest: Those with a family history of hypertension or those with hypertension had more experiences with blood pressure measurement in all analyses probably because they have an increased awareness of the health threats they might face.

⇒Thank you reviewer for your suggestion. Much appreciated. We improved the sentences as below.

Line350-356: Those with a family history of hypertension or those with hypertension had more experiences with blood pressure measurement in all analyses probably because they have an increased awareness of the health threats they might face. People with unhealthy lifestyle may not be aware of health threats or perceived health vulnerability, and one of the characteristics of people with healthy lifestyle may be the ability to perceive health threats, regardless of whether they have pre-existing health problems or not. People with unhealthy lifestyle (high salt intake or tobacco use) had less experience with blood pressure measurement.

Finally, there are still several grammar mistakes that I would suggest you proofread.

⇒We agree and therefore, we proofread again.

The authors declare no potential financial conflict of interest related to this manuscript. The funders played no role in the study design, data collection and analysis, decision to publish, or preparation of the manuscript.

We look forward to hearing from you at your earliest convenience.

Sincerely,

Yuichi Imanaka

Professor and Head,

Department of Health Care Economics and Quality Management

Graduate School of Medicine, Kyoto University, Japan 606-8501

Yoshida Konoe-cho, Sakyo-ku, Kyoto, Japan

Phone No: +81-75-753-4454 Fax No: +81-75-753-4455

Email Address: [imanaka-y@umin.net](mailto:imanaka-y@umin.net)
