# Supplementary material for: Social and behavioral factors related to blood pressure measurement: A cross-sectional study in Bhutan
Source: PLoS One. 2022 Aug 17;17(8):e0271914. doi: 10.1371/journal.pone.0271914 (PMC9385017; doi:10.1371/journal.pone.0271914)
Supplement: Attachment — Submitted filename: 20220620Response to Reviewers.docx [file pone.0271914.s006.docx]

PONE-D-21-12027R3

Social and behavioral factors related to blood pressure measurement: A cross-sectional study in Bhutan

PLOS ONE

We really appreciate your review and feedback.

Reviewer #2: In the abstract: Cardiovascular disease is a leading cause of death in the Kingdom of Bhutan, and the early detection of hypertension is critical for preventing it.

Again in the abstract clarify that “26% of the participants with hypertension never underwent blood pressure measurements” after their diagnosis since it is imposible to diagnose hypertension without a blood measurement, or whether this patients were identified during the STEPS survey.

⇒Thank you for your comment. We asked the English proofreader and had improved the sentence as follows.

Line53:“Approximately 26% of those with hypertension who were detected during the STEPS survey had never had their blood pressure measured.”

Line 59 says “having experienced with blood pressure measurement.” Check grammar.

⇒Thank you for your comment. We asked the English proofreader and had improved the sentence as follows.

Line60: “A family history of hypertension (OR: 2.019, 95% CI: 1.549–2.243) increased the likelihood of having experienced a blood pressure measurement in both men and women. “

Check grammar in lines 80-81, 215-216, 242, 243, 245, 253, 278, 300, 336.

⇒Thank you for your comments.

We have hired a professional English proofreader and improved some sentences.

The improved points were highlighted in green with you mentioning them, highlighted in yellow was without you mentioning them.

Line 238 would be better this way: Among those with hypertension, 26.3% (crude n=197, SE: 2.5%, 95% CI :21.7–31.4%) had never had blood pressure measurement before.

⇒Thank you for your advice. We had improved as follows.

Line257: “Among those with hypertension, 26.3% (crude n=197, SE: 2.5%, 95% CI :21.7–31.4%) had never had a blood pressure measurement before.

In line 268 you say Q1 was also significantly associated with the experience of blood pressure measurment, however Q1 was the reference so I do not understand this line.

We could find the significant association between Q3, Q4 and the experience of blood pressure measurement, but we could not find a significant association between Q2 (Nu 9,001–30,000: 120.9–402.9 USD) and the experience of blood pressure measurement.

⇒Thank you for your advice. We asked the English proofreader and had improved the sentence as follows.

Line 285: “In terms of income, all participants in Quartile-4 (Nu. 60,001+: USD 805.8+ (OR: 2.404, 95% CI: 1.575 –3.667)) and Quartile-3 (Nu. 30,001–60,000: USD 403.0–805.7 (OR: 2.546, 95% CI: 1.640–3.955)) had an increased likelihood of previous blood pressure measurement compared with those in the lowest income category, Quartile-1 (Nu. 0–9,000: USD 0–120.8 [OR: 1.000]). We found a significant association between Quartile-3, Quartile-4, and previous blood pressure measurement but not between Q2 (Nu. 9,001–30,000: USD 120.9–402.9) and previous blood pressure measurement.”

I highly recommend an English speaker proofreading since grammar mistakes have been present through revisions.

⇒Thank you for your advice. We asked the English speaker professional proofreader and had improved the whole sentence.
